# The Effect of Vitamin D Supplementation on Clinical Outcomes for Critically Ill Patients: A Systemic Review and Meta-Analysis of Randomized Clinical Trials

**DOI:** 10.3389/fnut.2021.664940

**Published:** 2021-05-04

**Authors:** Hejuan Shen, Yijun Mei, Kai Zhang, Xiaoya Xu

**Affiliations:** ^1^Department of General Surgery, Lishui People's Hospital, Lishui, China; ^2^Department of General Surgery, Sixth Affiliated Hospital of Wenzhou Medical University, Lishui, China; ^3^Department of Critical Care Medicine, Second Affiliated Hospital, Zhejiang University School of Medicine, Hangzhou, China

**Keywords:** vitamin D, critically ill, meta-analysis, intensive care unit, clinical outcome, nutrition

## Abstract

**Purpose:** Vitamin D deficiency is a common scenario in critically ill patients and has been proven to be associated with poor outcomes. However, the effect of vitamin D supplementation for critically ill patients remains controversial. Thus, we conducted a meta-analysis to evaluate the effect of vitamin D supplementation among critically ill patients.

**Methods:** Electronic databases PubMed, Embase, Scopus, and the Cochrane Library were searched for eligible randomized controlled trials between 2000 and January 2021. The primary outcome was overall mortality, and the secondary ones were the length of intensive care unit stay, the length of hospital stay, as well as the duration of mechanical ventilation. Subgroup analyses were performed to explore the treatment effect by type of admission, route of administration, dose of supplemented vitamin D, and the degree of vitamin D deficiency.

**Results:** A total of 14 studies involving 2,324 patients were finally included. No effect on overall mortality was found between vitamin D supplementation and control group [odds ratio (OR), 0.73; 95% CI, 0.52–1.03; *I*^2^ = 28%]. The vitamin D supplementation reduced the length of intensive care unit stay [mean difference (MD), −2.25; 95% CI, −4.07 to −0.44, *I*^2^ = 71%] and duration of mechanical ventilation (MD, −3.47; 95% CI, −6.37 to −0.57, *I*^2^ = 88%). In the subgroup analyses, the vitamin D supplementation for surgical patients (OR, 0.67; 95% CI, 0.47–0.94; *I*^2^ = 0%) or through parenteral way (OR, 0.42; 95% CI, 0.22–0.82, *I*^2^ = 0%) was associated with reduced mortality.

**Conclusion:** In critically ill patients, the supplementation of vitamin D has no effect on overall mortality compared to placebo but may decrease the length of intensive care unit stay and mechanical ventilation. Further trials are necessary to confirm our findings.

## Introduction

Vitamin D, a group of fat-soluble vitamins, plays a critical part in the regulation of bone metabolism and extraskeletal pleiotropic processes, such as immunomodulatory, antimicrobial, and cardiovascular (1, 2). Vitamin D deficiency is relevant to various disorders, including infections, diabetes, myocardial infarction, and autoimmune disease. The situation of the vitamin D deficiency occurs frequently not only in the general patients but also in critically ill patients (3, 4). Previous research indicated that the vitamin D status of critically ill patients often had a significant decrease during their intensive care unit (ICU) stay (5). The vitamin D deficiency in critically ill patients may result from a number of comorbidities, systemic inflammation, and multiorgan failure. Besides critical illness itself, some therapeutic interventions for critically ill patients including surgery, immobilization, fluid replacement, plasma exchange, hemodialysis filtration, and cardiopulmonary bypass may significantly reduce vitamin D levels (6). The incidence rate of vitamin D deficiency in critically ill patients is ranging from 26 to 82% (7, 8). More seriously, critically ill patients with vitamin D deficiency were accompanied by a series of poorer clinical consequences, such as higher possibility of nosocomial infections, increased susceptibility to sepsis, prolonged ICU or hospital stay, and increased overall mortality (9–13).

Considering the high incidence of vitamin D deficiency and its poor prognosis, the supplementation of vitamin D among critically ill patients has been proposed for many years. Vitamin D is best known for its role in the regulation of calcium levels through well-described gastrointestinal, renal, and bone actions. In addition, the vitamin D receptor has been identified on multiple other organs central to critical illness pathophysiology. Through these receptors, vitamin D exerts important physiological functions via both genomic and non-genomic pathways (5).

However, the effect of vitamin D supplementation for critically ill patients remains controversial (14–16). Several randomized controlled trials (RCTs) have suggested that vitamin D supplementation has a beneficial effect by decreasing the length of ICU and hospital stay, the duration of mechanical ventilation (MV), as well as the overall mortality rate (17–19). However, two RCTs with large sample sizes, the VITdAL-ICU and VIOLET trials, demonstrated that vitamin D supplementation had no additional benefits for critically ill patients (20, 21). However, the VITdAL-ICU trial (20) found that in the severe vitamin D deficiency subgroup, the usage of vitamin D supplementation results in a lower hospital mortality.

Therefore, the purpose of the study was to conduct an updated meta-analysis of all RCTs to assess the effect of vitamin D supplementation for clinical outcomes of critically ill patients.

## Methods

### Data Sources and Study Selection

We followed the guidelines of the Cochrane handbook methodology (22) and PRISRMA statement (23) (checklist in Supplementary Material 1) to perform this meta-analysis. The study protocol was registered in PROSPERO (CRD 42020169411). We searched PubMed, Embase, Scopus, EBSCO, and Cochrane Library for eligible studies between 2000 and January 10, 2021. The literature search was confined to articles written only in English. The detailed search strategies were recorded in Supplementary Material 2.

### Eligibility Criteria

Study inclusion criteria were as follows: (1) population—critically ill adult patients (≥18 years of age), defined as patients admitted to an ICU or received intensive care measures (e.g., MV); (2) intervention—vitamin D supplementation through enteral or parenteral route; (3) comparison—placebo or no drug infusion; (4) outcomes—the primary outcome was overall mortality, including ICU, hospital, and 28-day mortality, and secondary outcomes were ICU and hospital length of stay (LOS) and duration of MV; and (5) design—RCT.

### Data Extraction and Quality Assessment

Two authors independently retrieved and derived relevant studies. The basic characteristics of included studies (first author, years of publication, population, intervention and control methods, vitamin D level) are recorded in Table 1. Some detailed information like study design, sample size, sex ratio, mean age, and inclusion and exclusion criteria were recorded in Supplementary Material 3. Any discrepancies in all phases were ultimately resolved through team consensus.

**Table 1:** Characteristics of studies included in the meta-analysis.

**Study**	**Sample size**	**Population**	**Interventions**	**Vitamin D level**	
				**Vitamin D group**	**Placebo group**
Naguib et al. (24)	86 (vitamin D, 45; placebo, 41)	Patients undergoing valve replacement surgery	Oral dose of 2 μg/day alfacalcidol started 48 h before surgery and continued throughout the hospital stay	Baseline: 21.0 ± 11.2 ng/ml; Day 3: 23.4 ± 10.6 ng/ml	Baseline: 19.1 ± 9.5 ng/ml; Day 3: 16.5 ± 8.0 ng/ml
Sharma et al. (25)	35 (vitamin D, 20; placebo, 15)	Acute traumatic brain injury patients	Oral dose of 120,000 IU vitamin D3 or placebo for 14 days	Baseline: 18.3 (14.5–23.0) ng/ml; Day 14: 39.2 (36.8–44.6) ng/ml	Baseline: 15.2 (11.8–26.9) ng/ml; Day 14: 27.3 (14.6–30.8) ng/ml
Ingels et al. (26)	24 (vitamin D, 11; placebo, 13)	Critically ill patients in SICU	An IV loading dose of 200 μg and maintenance dose of 15 μg vitamin D3 per day, or IV injection of placebo for 10 days	Baseline: 9.2 (7.2–13.1) ng/ml; Day 10: about 16 ng/ml	Baseline: 6.8 (5.1–10.2) ng/ml; Day 10: about 8 ng/ml
Ginde et al. (21)	1,078 (vitamin D, 538; placebo, 540)	Critically ill patients, more than 80% patients were medical patients	Single enteral does of 540,000 IU of vitamin D3 or placebo for 90 days	Baseline: 11.2 ± 4.8 ng/ml; Day 3: 46.9 ± 23.2 ng/ml	Baseline: 11.0 ± 4.7 ng/ml; Day 3: 11.4 ± 5.6 ng/ml
Miri et al. (27)	40 (vitamin D, 22; placebo, 18)	Mechanically ventilated, adult ICU patients	Intramuscular injection of 300,000 IU vitamin D3 or placebo for 14 days	Baseline: 8.4 ± 6.8 ng/ml; Day 7: 10.5 ± 9.8 ng/ml	Baseline: 11.4 ± 18.2 ng/ml; Day 7: 11.2 ± 18.2 ng/ml
Karsy et al. (28)	267 (vitamin D, 134; placebo, 133)	Neurocritical care patients	Single enteral does of 540,000 IU of vitamin D3 or placebo for 30 days	Baseline: 14.6 ± 4.2 ng/ml; Day 3: 20.8 ± 9.3 ng/ml	Baseline: 13.9 ± 4.6 ng/ml; Day 3: 12.8 ± 4.8 ng/ml
Hasanloei et al. (18)	72 (oral vitamin D, 24; injection vitamin D, 24; control, 24)	Traumatic mechanical ventilated patients	Oral dose of 50,000 IU vitamin D3 daily or intramuscular injection of 300,000 IU vitamin D3 for 6 days, no placebo	Oral group: Baseline: 17.1 ± 4.5 ng/ml; After intervention: 28.6 ± 4.0 ng/ml; Injection group: baseline: 18.7 ± 3.3 ng/ml; After intervention: 29.4 ± 5.2 ng/ml	Baseline: 17.0 ± 3.3 ng/ml; After intervention: 16.1 ± 2.7 ng/ml
Parekh et al. (29)	68 (vitamin D, 33; placebo, 35)	ICU patients after elective esophagectomy	Single oral preoperative (3–14 days) dose of 300,000 vitamin D3 or placebo	Baseline: 19.0(12.8–27.1) ng/ml; Preoperative: 29.9(25.4–37.0) ng/ml Postoperative day 3: 22.0(17.3–27.8) ng/ml	Baseline: 18.5(14.2–27.6) ng/ml; Preoperative: 17.1(13.0–23.4) ng/ml Postoperative day 3: 11.2(7.8–16.2) ng/ml
Miroliaee et al. (30)	46 (vitamin D, 24; placebo, 22)	Patients with ventilator-associated pneumonia	Intramuscular injection of 300,000 IU vitamin D3 or placebo for 28 days	Baseline: 17.1 ± 6.1 ng/ml; The vitamin D level increased 12.3 ± 8.3 ng/ml after 7 days	Baseline: 19.5 ± 4.6 ng/ml; The vitamin D level increased 1.2 ± 1.5 ng/ml after 7 days
Han et al. (17)	30 (low-dose vitamin D, 9; high-dose vitamin D, 11; placebo, 10)	Mechanically ventilated patients, 16 in SICU and 14 in MICU	Low-dose vitamin D group received 50,000 IU of vitamin D3 daily for 5 days; High-dose vitamin D group received 100,000 IU of vitamin D3 daily for 5 days; Control group received placebo daily for 5 days	Low-dose vitamin D group: Baseline: 23.2 ± 7.8 ng/ml; Day 7: 45.0 ± 20.0 ng/ml High-dose vitamin D group: Baseline: 20.0 ± 7.3 ng/ml; Day 7: 55.0 ± 14.0 ng/ml	Baseline: 21.5 ± 12.2 ng/ml; Day 7: NR
Quraishi et al. (19)	30 (low-dose vitamin D, 10; high-dose vitamin D, 10; placebo, 10)	Patients with sepsis, 16 in MICU and 14 in SICU	Low-dose vitamin D group received 200,000 IU of vitamin D3 daily; High-dose vitamin D group received 400,000 IU of vitamin D3 daily; Control group received placebo daily	Low-dose vitamin D group: Day 1: 15 (12–20) ng/ml; Day 5: 22 (16–25) ng/ml High-dose vitamin D group: Day 1: 17 (13–25) ng/ml; Day 5: 29 (23–41) ng/ml	Day 1: 19 (13–22) ng/ml; Day 5: 19 (11–23) ng/ml
Amrein et al. (20)	475 (vitamin D, 237; placebo, 238)	Critically ill patients, more than 75% patients were surgical or neurologic patients	Vitamin D3 or placebo was given orally or via nasogastric tube once at a dose of 540,000 IU followed by monthly maintenance doses of 90,000 IU for 5 months	Baseline: 13.0 ± 4.0 ng/ml; Day 3: 33.5 ± 18.7 ng/ml; Day 7: 35.5 ± 20.6 ng/ml	Baseline: 13.1 ± 4.3 ng/ml; Day 3: 13.9 ± 5.0 ng/ml; Day 7: 14.5 ± 5.1 ng/ml
Leaf et al. (31)	67 (vitamin D, 36; placebo, 31)	Patients with severe sepsis or septic shock, 38 in SICU and 29 in MICU	Single intravenous dose of calcitriol, 2 mg, or equal volume of saline	Baseline: 14.1 (9.3–36.4) pg/ml; 6 h: 75.7 (52.1–115.5) pg/ml	Baseline: 13.7 (10.7–30.8) pg/ml; 6 h: 16.9 (9.0–26.9) pg/ml
Amrein et al. (32)	25 (vitamin D, 12; placebo, 13)	Critically ill patients in MICU	540,000 IU of vitamin D3 or placebo orally or via feeding tube	Baseline: 13.1 ng/ml; Day 3: 33.1 ng/ml; Day 7: 38.2 ng/ml	Baseline: 14.1 ng/ml; Day 3: 15.0 ng/ml; Day 7: 13.7 ng/ml

*IU, international unit; SICU, surgical intensive care unit; IV, injection of vein; MICU, medical intensive care unit; ICU, intensive care unit*.

Two authors evaluated the risk of bias independently according to the Cochrane risk of bias tool (33). The details for quality assessment were recorded in Supplementary Material 3.

### Statistical Synthesis and Analysis

We presented results as odds ratio (OR) with 95% confidence interval (CI) for dichotomous data and mean difference (MD) with 95% CI for continuous data. We tested heterogeneity between studies by the chi-squared test with significance set at *P*-value of 0.1 and quantitatively by inconsistency (*I*^2^) statistics (34). Significant heterogeneity was suggested when *I*^2^-value >50%. In consideration of the significant difference in sample size between Ginde et al. (21) and the other studies, a random effect model was employed to perform the analysis. In addition, we adopted the funnel plot and Egger's regression test to investigate the potential publication bias. If one trial contained more than two cohorts, we combined the data according to the recommendation in Cochrane handbook.

Subgroup analyses were performed to assess the possible influence on the outcomes of the type of admission (surgical vs. non-surgical patients), route of administration (enteral vs. parenteral administration), dose of supplemented vitamin D, and the degree of vitamin D deficiency (severe vs. less severe). The threshold of high-dose vitamin D administration was set to 300,000 IU daily according to the review of Kearns et al. on vitamin D supplementation in adult (35), and severe vitamin D deficiency was defined as vitamin D level <12.5 ng/ml at study inclusion (20).

Furthermore, a sensitivity analysis was employed to examine the effect of individual study by omitting each one at a time.

## Results

### Study Characteristics

**Figure 1:**
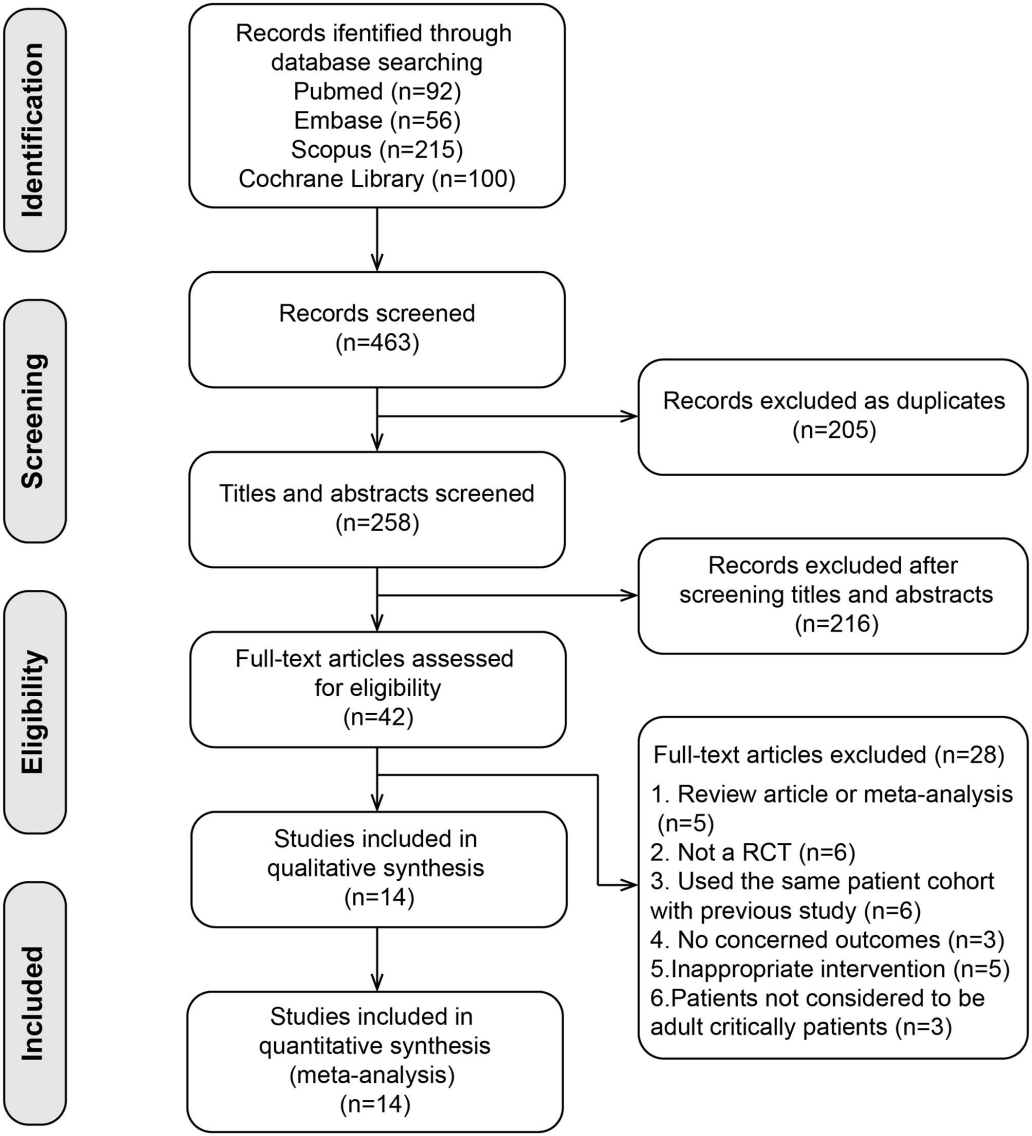
Flow diagram for the selection of studies.

A total of 463 relevant articles were initially searched. We identified 42 studies after removing duplicates and screening abstracts. Among them, 28 studies were further excluded in the full-text assessments (list of excluded studies with reasons in Supplementary Material 4). Finally, we included 14 studies (17–21, 24–32) involving 2,324 patients in our meta-analysis (flow diagram in Figure 1). The sample size ranged from 25 to 1,078, including 10 small sample studies (number of included patients <100) (17–19, 24–27, 29–32). The analyzed population included patients with various disorders, including medical patients (21, 30, 32), neurological or surgical patients (18, 20, 24–26, 28, 29), and both medical and surgical patients (17, 19, 27, 31). In 10 trials, all the participants had vitamin D deficiency (≤20 ng/ml) (18, 20, 21, 25–30, 32). In the trial conducted by Naguib et al. (24), about 60% of the participants had vitamin D deficiency. Han et al. (17) included critically ill patients with MV; 43% of them had vitamin D deficiency. Two studies included sepsis patients, and the number of patients with vitamin D deficiency was not specified (19, 31). Almost all studies administered vitamin D_3_ (cholecalciferol), and two studies administered calcitriol (24, 31). Vitamin D was administered through oral or enteral route in eight studies (17, 19–21, 24, 25, 28, 29, 32), through parenteral route such as intramuscular or intravenous injection in four (26, 27, 30, 31), and both route in one (18). Vitamin D supplementation could restore the plasma vitamin D concentration in all the trials. The serum level of vitamin D was significantly higher in the intervention group compared with the control group except the trial by Miri et al. (27).

### Quality Assessment

The risk of bias assessment was summarized in Supplementary Figure 1. Three studies were rated as high risk of bias: Hasanloei et al. (18) did not use blinding method; in the study by Miri et al. (27), the condition of vitamin D deficiency did not get significant improvement after 7 days intervention; Parekh et al. (29) administered vitamin D before patients entering ICU. The details for quality assessment and reason for judgment were reported in Supplementary Material 3.

The test of asymmetry on the funnel plot and Egger's test was concluded for every outcome. We observed potential publication bias for the primary outcome (Supplementary Figure 2A, Egger's test: *P* < 0.05); thus, we performed an analysis using the trim and fill method. After imputing, the funnel plot became symmetrical (Supplementary Figure 2B), and the pooled estimate continued to show no association between vitamin D supplementation and overall mortality (OR, 0.97; 95% CI, 0.69–1.38, *I*^2^ = 37%). For the second outcomes, no significant publication bias was observed for length of ICU (Egger's test: *P* = 0.23, e-Figure 2c) and hospital stay (Egger's test: *P* = 0.79, e-Figure 2d). For the duration of MV, the funnel plot and Egger's test (*P* = 0.06, e-Figure 2e) indicated that there was potential publication bias, and the analysis after imputing showed no significant difference between groups (MD, −0.25; 95% CI, −2.80–2.30, *I*^2^ = 91%, e-Figure 2f).

### Primary Outcome

**Figure 2:**
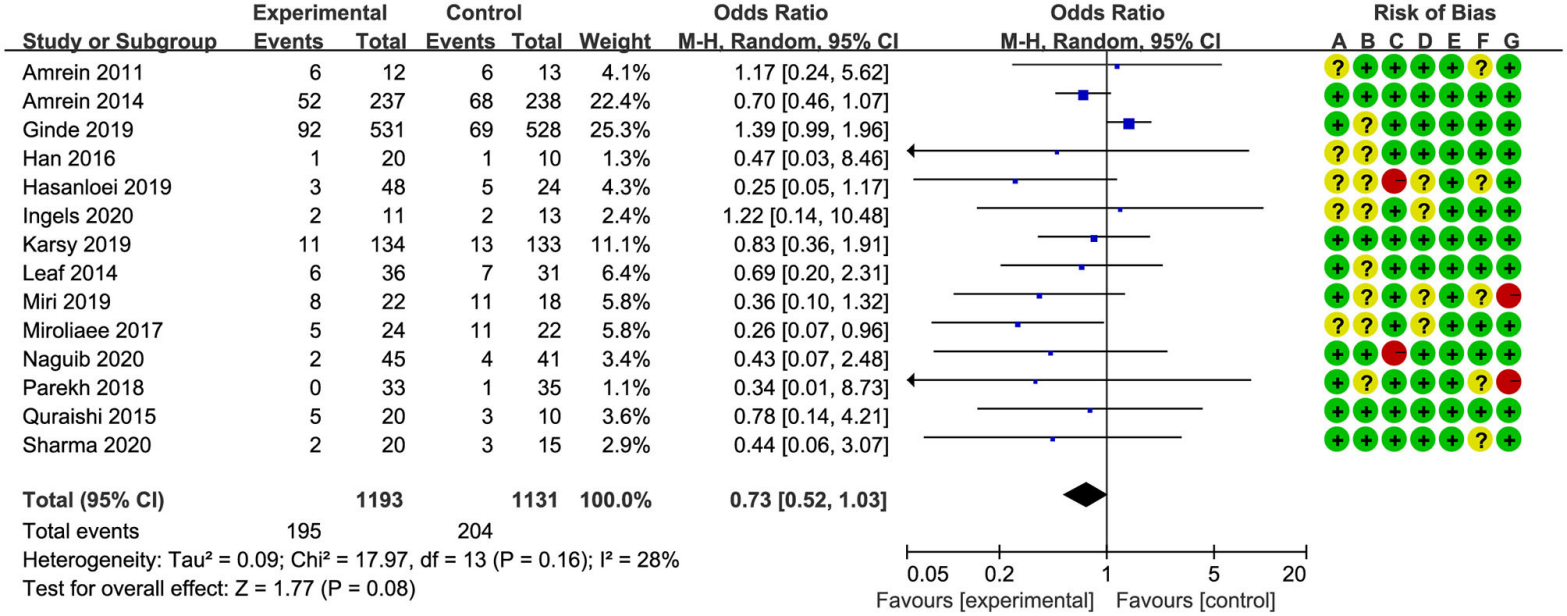
Effect of vitamin D administration on mortality in critically ill patients [risk of bias: (A) random sequence generation; (B) allocation concealment; (C) blinding of participants and personnel; (D) blinding of outcome assessment; (E) incomplete outcome data; (F) selective reporting; (G) other bias].

Overall mortality was screened with different measures in all studies. Six studies (17, 18, 24–26, 32) reported in-hospital mortality, four studies (19, 27, 28, 30) reported 28-/30-day mortality, and four studies (20, 21, 29, 31) reported multiple results; we chose 28-/30-day mortality in the analysis. The pooled result indicated that vitamin D supplementation did not reduce overall mortality rate for critically ill patients (OR, 0.73; 95% CI, 0.52–1.03; *I*^2^ = 28%, Figure 2). In addition, four studies reported long-term mortality, and the vitamin D supplementation did not improve long-term survival rate as well (OR, 0.95; 95% CI, 0.69–1.31; *I*^2^ = 26%, Supplementary Figure 3).

### Secondary Outcomes

**Figure 3:**
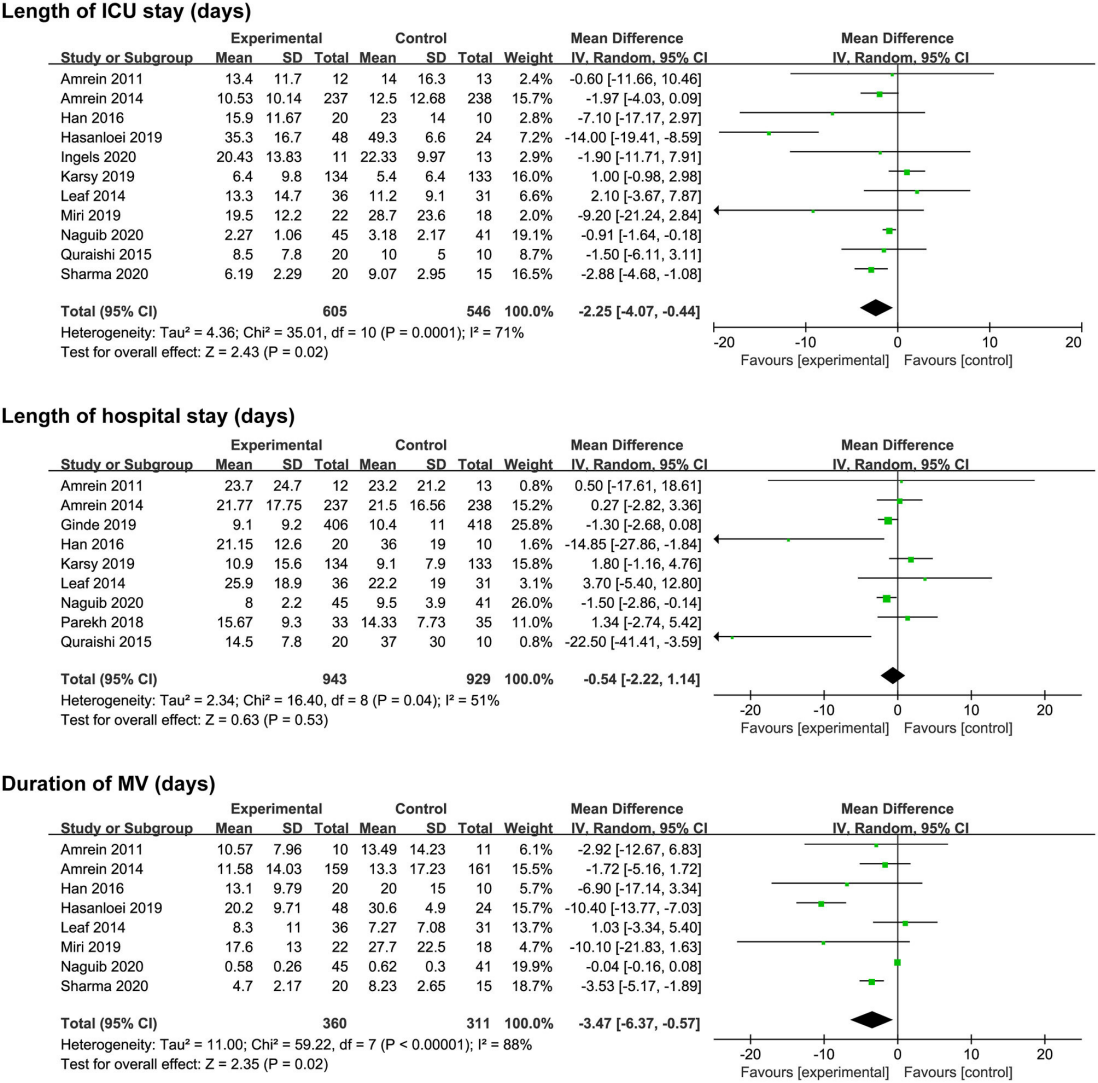
Effect of vitamin D administration on length of ICU stay, length of hospital stays, and duration of MV. ICU, intensive care unit; MV, mechanical ventilation.

Eleven studies (17–20, 24–28, 31, 32) reported length of ICU stay, nine (17, 19–21, 24, 28, 29, 31, 32) reported length of hospital stay, and eight reported (17, 18, 20, 24, 25, 27, 31, 32) duration of MV. The vitamin D supplementation was associated with a reduction in length of ICU stay (MD, −2.25; 95% CI, −4.07 to −0.44; *I*^2^ = 71%; Figure 3) and duration of MV (MD, −3.47; 95% CI, −6.37 to −0.57; *I*^2^ = 88%; Figure 3). However, there was no significant difference in length of hospital stay between the two groups (MD, −0.54; 95% CI, −2.22 to 1.14; *I*^2^ = 51%; Figure 3). Furthermore, significant heterogeneity was on the limit for these analyses.

### Sensitivity Analysis

We analyzed the effect of every single trial on the pooled result by omitting each study. The supplementation of vitamin D was relevant to the obvious decreasing in overall mortality after omitting the study by Ginde et al. (21) (Supplementary Figure 4). For the secondary outcomes, the reduction in length of ICU stay became not statistically significant when omitting some studies, indicating the poor robustness. The other two outcomes showed no significant differences during this analysis (Supplementary Figure 5).

### Subgroup Analysis

We performed subgroup analyses to assess whether the type of admission, route of supplementation, dose of supplemented vitamin D, and degree of vitamin D deficiency would affect the clinical outcomes. The results of subgroup analyses are shown in Table 2.

**Table 2:** Main findings and subgroup analysis.

**Analyses**	**Subgroup**	**Effect estimate (95%CI), heterogeneity**	
**Mortality**	Total	OR 0.73 (0.52, 1.03), *I*^2^ = 28% (*P =* 0.16)	
Type of admission	Surgical	OR 0.67 (0.47, 0.94), *I*^2^ = 0% (*P =* 0.84)	Subgroup difference *I*^2^ = 0% (*P =* 0.78)
	Non-surgical	OR 0.74 (0.40, 1.35), *I*^2^ = 43% (*P =* 0.10)	
Administration route	Enteral	OR 0.91 (0.67, 1.23), *I*^2^ = 13% (*P =* 0.32)	Subgroup difference *I*^2^ = 77% (*P =* 0.04)
	Parenteral	OR 0.42 (0.22, 0.82), *I*^2^ = 0% (*P =* 0.59)	
Vitamin D dose	<300,000 IU	OR 0.55 (0.27, 1.12), *I*^2^ = 0% (*P =* 0.97)	Subgroup difference *I*^2^ = 0% (*P =* 0.46)
	≥300,000 IU	OR 0.75 (0.51, 1.11), *I*^2^ = 39% (*P =* 0.09)	
Vitamin D deficiency	Severe	OR 0.73 (0.32, 1.68), *I*^2^ = 75% (*P < * 0.01)	Subgroup difference *I*^2^ = 0% (*P =* 0.98)
	Less-severe	OR 0.72 (0.50, 1.03), *I*^2^ = 0% (*P =* 0.74)	
**ICU LOS**	Total	MD −2.25 (−4.07, −0.44), *I*^2^ = 71% (*P* < 0.01)	
Type of admission	Surgical	MD −2.47 (−4.65, −0.29), *I*^2^ = 84% (*P <* 0.01)	Subgroup difference *I*^2^ = 0% (*P =* 0.64)
	Non-surgical	MD −1.51 (−4.84, 1.81), *I*^2^ = 6% (*P =* 0.37)	
Administration route	Enteral	MD −2.16 (−3.96, −0.37), *I*^2^ = 73% (*P <* 0.01)	Subgroup difference *I*^2^ = 35% (*P =* 0.22)
	Parenteral	MD −5.45 (−10.34, −0.56), *I*^2^ = 67% (*P =* 0.03)	
Vitamin D dose	<300,000 IU	MD −3.71 (−6.67, −0.75), *I*^2^ = 81% (*P <* 0.01)	Subgroup difference *I*^2^ = 0% (*P =* 0.52)
	≥300,000 IU	MD −2.40 (−5.14, 0.34), *I*^2^ = 60% (*P <* 0.01)	
Vitamin D deficiency	Severe	MD −1.27 (−4.09, 1.56), *I*^2^ = 0% (*P =* 0.40)	Subgroup difference *I*^2^ = 0% (*P =* 0.68)
	Less-severe	MD −1.98 (−3.90, −0.07), *I*^2^ = 68% (*P <* 0.01)	
**Hospital LOS**	Total	MD −0.54 (−2.22, 1.14), *I*^2^ = 51% (*P =* 0.04)	
Type of admission	Surgical	MD −0.16 (−2.23, 1.90), *I*^2^ = 55% (*P =* 0.11)	Subgroup difference *I*^2^ = 0% (*P =* 0.49)
	Non-surgical	MD −1.83 (−6.09, 2.43), *I*^2^ = 0% (*P =* 0.48)	
Administration route	Enteral	MD −0.68 (−2.39, 1.04), *I*^2^ = 55% (*P =* 0.03)	Subgroup difference *I*^2^ = 0% (*P =* 0.35)
	Parenteral	MD 3.70 (−5.40, 12.80), Not applicable	
Vitamin D dose	<300,000IU	MD −11.18 (−24.95, 2.59), *I*^2^ = 79% (*P <* 0.01)	Subgroup difference *I*^2^ = 60% (*P =* 0.11)
	≥300,000IU	MD 0.10 (−1.96, 2.15), *I*^2^ = 37% (*P =* 0.15)	
Vitamin D deficiency	Severe	MD −0.17 (−3.61, 3.27), *I*^2^ = 52% (*P =* 0.15)	Subgroup difference *I*^2^ = 0% (*P =* 0.86)
	Less-severe	MD −0.56 (−3.10, 1.98), *I*^2^ = 55% (*P =* 0.03)	
**Duration of MV**	Total	MD −3.47 (−6.37, −0.57), *I*^2^ = 88% (*P <* 0.01)	
Type of admission	Surgical	MD −3.70 (−7.35, −0.05), *I*^2^ = 94% (*P <* 0.01)	Subgroup difference *I*^2^ = 0% (*P =* 0.79)
	Non-surgical	MD −2.86 (−7.94, 2.22), *I*^2^ = 33% (*P =* 0.21)	
Administration route	Enteral	MD −3.83 (−7.02, −0.64), *I*^2^ = 90% (*P <* 0.01)	Subgroup difference *I*^2^ = 0% (*P =* 0.69)
	Parenteral	MD −5.70 (−14.20, 2.80), *I*^2^ = 83% (*P <* 0.01)	
Vitamin D dose	<300,000 IU	MD −4.60 (−8.68, −0.53), *I*^2^ = 94% (*P <* 0.01)	Subgroup difference *I*^2^ = 0% (*P =* 0.85)
	≥300,000 IU	MD −4.01 (−8.62, 0.60), *I*^2^ = 70% (*P =* 0.01)	
Vitamin D deficiency	Severe OR	MD −10.10 (−21.83, 1.63), Not applicable	Subgroup difference *I*^2^ = 21% (*P =* 0.26)
	Less-severe	MD −3.14 (−6.08, −0.20), *I*^2^ = 89% (*P <* 0.01)	

*ICU, intensive care unit; LOS, length of stay; MV, mechanical ventilation; OR, odds ratio; MD, mean difference; CI, confidence interval; IU, international unit; OR, odds ratio; MD, mean difference*.

**Figure 4:**
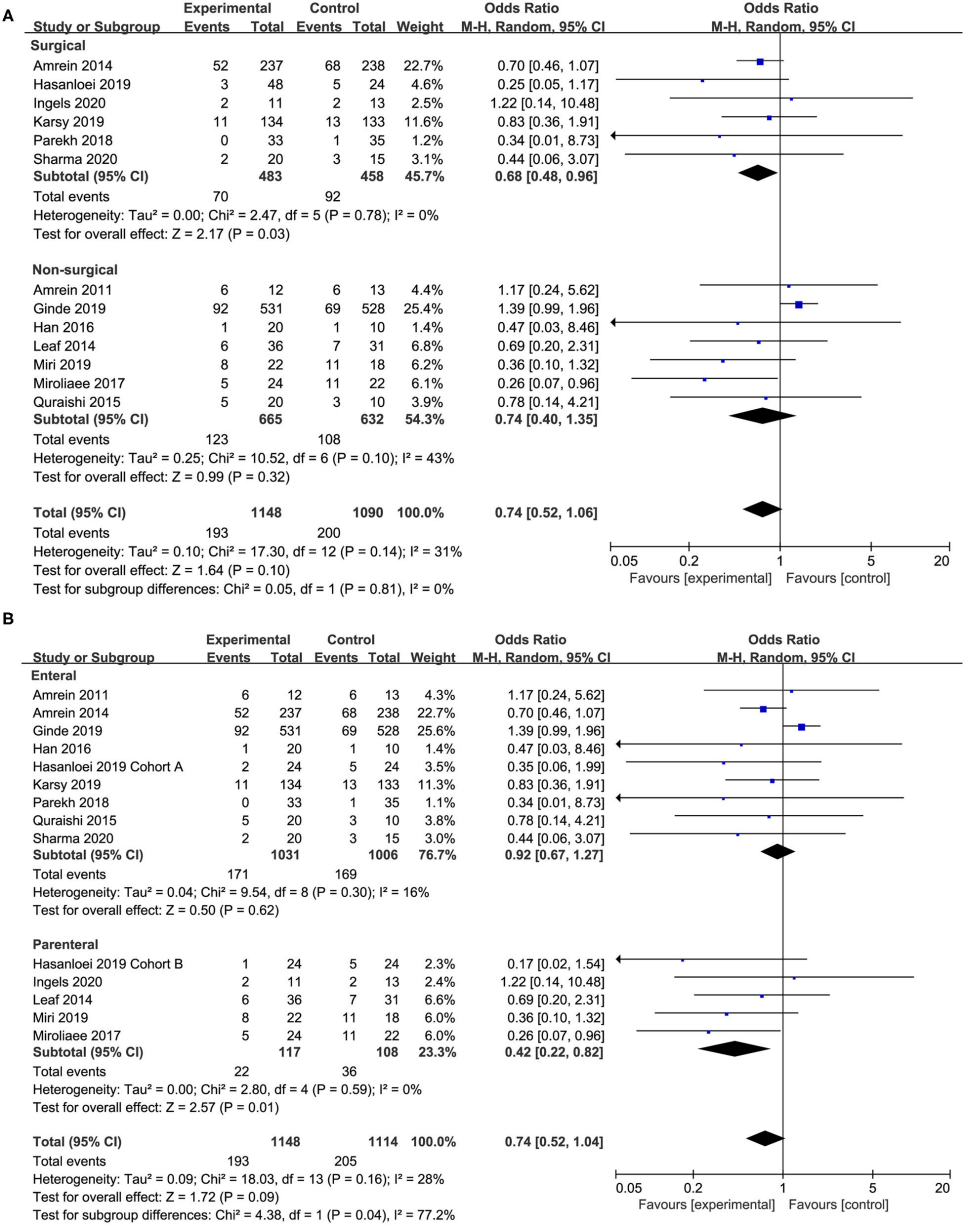
Subgroup analysis for primary outcome. **(A)** Surgical vs. non-surgical patients; **(B)** enteral vs. parenteral route.

Seven studies (18, 20, 24–26, 28, 29) compared the effect of vitamin D supplementation in surgical patients, and we found the supplementation of vitamin D was associated with a reduced mortality rate (OR, 0.67; 95% CI, 0.47–0.94; *I*^2^ = 0%; Figure 4A) in this specific group. However, the non-surgical group showed no significant improve in mortality reduction (OR, 0.74; 95% CI, 0.40–1.35; *I*^2^ = 43%; Figure 4A). Furthermore, there was no significant difference between these two subgroups regarding length of ICU or hospital stay and duration of MV (Supplementary Figures 7–9).

In 10 of the included studies, vitamin D was administered by enteral route (17–21, 24, 25, 28, 29, 32) while five studies by parenteral (18, 26, 27, 30, 31). The enteral subgroup showed no improvement in mortality reduction (OR, 0.91; 95% CI, 0.67–1.23; *I*^2^ = 13%; Figure 4B). When analyzing trials administered parenteral vitamin D, we found a reduction in overall mortality (OR, 0.42; 95% CI, 0.22–0.82; *I*^2^ = 0%; Figure 4B). The different administration route has no significant influence on the length of ICU or hospital stay and the duration of MV (Supplementary Figures 7–9).

In addition, we also explored the effect of vitamin D dose and degrees of vitamin D deficiency on the clinical outcomes. In six studies (17–19, 24, 25, 31), the dose of vitamin D was relatively low (<300,000 IU daily). In four studies (20, 21, 26, 27), enrolled patients were defined as having severe vitamin D deficiency at study inclusion (vitamin D <12.5 ng/ml). However, the subgroup analyses showed that there was no significant difference between subgroups (Supplementary Figures 6–9).

## Discussion

In this meta-analysis, we included 14 studies with 2,324 patients to analyze the effect of vitamin D supplementation in critically ill patients. The preliminary analysis showed that the vitamin D supplementation group had better outcomes than the control group from the numerical perspective, which reveals that vitamin D supplementation has potential clinical benefits. However, there is no significance for overall mortality and length of hospital stay from the statistical perspective. The obvious heterogeneity was limited for other outcomes (length of hospital stay and duration of MV). But interestingly, we found that vitamin D supplementation reduced the overall mortality in surgical patients, and vitamin D supplementation through parenteral way was associated with reduced mortality as well. In addition, both enteral and parenteral administration are effective in increasing vitamin D blood level compared with placebo, without major adverse events.

To our knowledge, this is the most updated and comprehensive meta-analysis to evaluate the effect of vitamin D supplementation in critically ill patients. Our results are generally consistent with the latest meta-analysis by Lan et al. (16), in which they analyzed nine RCTs with 1,867 patients. They indicated that vitamin D supplementation did not reduce the mortality, length of ICU and hospital stay, as well as duration of MV. However, our results demonstrated that the vitamin D supplementation reduced the length of ICU stay and duration of MV. This difference resulted from several newly published RCTs (24–26, 28, 29). Compared with previous meta-analysis, our study included more updated RCTs, more critically ill patients, and more subgroup analyses. In addition, we found that vitamin D supplementation was able to reduce the overall mortality rate of surgical patients. What is more, the overall mortality rate can be decreased through the parenteral way.

The possible mechanisms of the benefit of vitamin D supplementation in critically ill patients can be explained in several ways. First, the vitamin D supplementation in critically ill patients would restore the plasma vitamin D concentration. All the trials analyzed in our meta-analysis reported an increased serum level of vitamin D in the intervention group. Second, vitamin D regulates the expression of the antimicrobial peptides cathelicidin and β-defensin, both of which have functional effectors within the immune system (36). Cathelicidin can upregulate the levels of anti-inflammatory cytokines and downregulate the production of proinflammatory cytokines in response to Gram-negative bacteria, positive bacteria, and fungi (19, 37). Thus, vitamin D deficiency may increase the risk of inflammation and sepsis in the critically ill by the suppression of immune reactivity and stimulatory effects on innate immunity (38, 39). To this end, we made an additional analysis about the incidence of infection between the vitamin D supplementation group and control group. The result demonstrated that critically ill patients receiving vitamin D had a lower infection rate (OR, 0.70; 95% CI, 0.51–0.98; *I*^2^ = 0%; Supplementary Figure 10). Third, vitamin D affects the modulation of bone and muscle metabolism (40, 41). Vitamin D deficiency may contribute to impaired bone formation, bone hyperresorption, and skeletal muscle dysfunction, which may further lead to delayed wound healing, difficult weaning or weaning failure, and prolonged ICU and hospital stay.

But in our meta-analysis, as suggested by the negative result of primary outcome, the survival benefit does not happen. The mechanisms of the benefit of vitamin D supplementation in critically ill patients based on the intervention would restore the plasma vitamin D concentration and then improve the clinical outcomes. However, there may be a lag between vitamin D administration and observing clinical benefit. Therefore, we hypothesized that the time to await these vitamin D actions to occur is not sufficient for some more severely ill patients, as the trajectory of acute illness that finally leads to multiorgan dysfunction and death has already commenced. In other words, some critically ill patients died too early, and there was not enough time for effective vitamin D supplementation. In a *post hoc* analysis from the VITDAL-ICU study, researchers found that the vitamin D supplementation was associated with a reduction of 28-day mortality after excluding the early dead or discharged participants within the first 7 days (42). Therefore, we suggest that further trials should consider excluding patients with very high baseline severity scores or who die in the early course of the ICU stay or at least to plan in advance subgroup analyses excluding patients with early deaths and early discharge. Even if these analyses did not reach statistical significance, the results are of notable interest. A modest benefit may be clinically relevant, and further large RCTs are warranted.

In subgroup analyses, we compared the clinical outcomes between parenteral and oral/enteral route of vitamin D supplementation. As we know, vitamin D becomes a biologically active hormone after forming 25-OH vitamin D in the liver and calcitriol (1,25-OH vitamin D) in the kidney (43). Vitamin D levels are affected by many factors, such as vitamin D intake, absorption and adiposity (44). More importantly, the levels of vitamin D are affected by acute kidney injury, infection, fluid overload, and immobilization, which occur frequently in critically ill patients. Therefore, compared with oral/enteral route, parenteral administration can increase the vitamin D concentration in serum much easier. This is why vitamin D administration through parenteral way can reduce mortality compared with the enteral route.

When we analyzed the effect of every single trial on the pooled results by omitting each study, we found that after omitting the study by Ginde et al. (12), vitamin D supplementation reduced overall mortality significantly. In contrast, our current data showed that vitamin D supplementation improved the mortality rate in the surgical subgroup but not overall mortality in critically ill patients. Ginde's study primarily enrolled typical medical patients in the ICU, such as patients with pneumonia, respiratory failure, and sepsis, which is the most recently large sample study. Considering high risk of imprecision bias, more results and evidence about the effects of vitamin D supplementation on surgical and medical patients are compellingly needed in the future.

However, our study has several limitations. First of all, although we focused on critically ill patients, the studied population was very broad and heterogeneous. For example, some were admitted to the surgical ICU after a major operation; some had severe traumatic injury. Some trials enrolled typical medical ICU patients with pneumonia, respiratory failure, shock, or sepsis. We assumed the vitamin D might have a different effect on subgroups of this broad population. The subgroup analysis showed that vitamin D supplementation could reduce the mortality in the surgical subgroup. Similarly, the clinical characteristics of included studies were heterogeneous. The baseline of vitamin D level, dose and route of vitamin D supplementation, as well as the disease severity of enrolled patients are varied across all the studies. Thus, the pooled estimates should be interpreted with caution due to the significant heterogeneity.

Moreover, hypercalcemia is the most common adverse effect when people receive high-dose vitamin D (45). Considering only a few articles reported hypercalcemia as a vitamin-D-related adverse event, there was not enough data to evaluate the incidence of hypercalcemia between vitamin D supplementation and control groups. Finally, the sample size in some included trials was relatively small (number of participants <100), which may introduce small-study effects and get larger beneficial treatment effects conclusion (46).

## Conclusion

In this most updated and comprehensive meta-analysis, we found that vitamin D supplementation has no effect on overall mortality in critically ill patients but was associated with a significant reduction in length of ICU stay and duration of MV. Although the statistical heterogeneity worsened the strength of inference with respect to the benefits on clinical outcomes of vitamin D supplementation, the observed favorable effects of vitamin D supplementation on reducing overall mortality in surgical patients and the effectiveness of parenteral administration on increasing vitamin D blood levels should be considered. More RCTs and further research are needed to confirm the potential benefits of vitamin D supplementation in critically ill patients.

## Data Availability Statement

The raw data supporting the conclusions of this article will be made available by the authors, without undue reservation.

## Author Contributions

HS and XX contributed to the acquisition and analysis of the data and the initial draft writing of this paper. HS, KZ, and YM contributed to the collection and interpretation of data. XX contributed to the concept of the review, the revision of this paper, and the final approval of the version to be published. All authors contributed to the article and approved the submitted version.

## Conflict of Interest

The authors declare that the research was conducted in the absence of any commercial or financial relationships that could be construed as a potential conflict of interest.
