# Supplementary material for: The Effect of Vitamin D Supplementation on Clinical Outcomes for Critically Ill Patients: A Systemic Review and Meta-Analysis of Randomized Clinical Trials
Source: Front Nutr. 2021 May 4;8:664940. doi: 10.3389/fnut.2021.664940 (PMC8129506; doi:10.3389/fnut.2021.664940)
Supplement: Supplementary file 2 [file Data_Sheet_2.PDF]

Additional file 2: Search strategies

Pubmed

#1 cholecalciferol[Title/Abstract] OR vitamin D[Title/Abstract] OR vitamin D[MeSH Terms] OR 25-hydroxyvitamin D[Title/Abstract] OR calcitriol [Title/Abstract] OR 1,25-dihydroxyvitamin D [Title/Abstract]

#2 random*[Title/Abstract] OR "Randomized Controlled Trial"[pt]

#3 intensive care units [MeSH Terms] OR critical care [MeSH Terms] OR critical care [Title/Abstract] OR critical illness[MeSH Terms] OR critical illness [Title/Abstract] OR critically ill [Title/Abstract] OR intensive care [Title/Abstract] OR ICU [Title/Abstract]

#1 AND #2 AND #3

Embase

#1 (vitamin D):ti,ab,kw OR (cholecalciferol):ti,ab,kw OR (hydroxyvitamin D):ti,ab,kw OR (calcitriol):ti,ab,kw OR (dihydroxyvitamin D):ti,ab,kw

#2 (icu):ti,ab,kw OR (critical care):ti,ab,kw OR (critical illness):ti,ab,kw OR (critically ill):ti,ab,kw OR (intensive care):ti,ab,kw

#3 (randomized controlled trial):ti,ab,kw

#1 AND #2 AND #3

Cochrane Library

#1 vitamin d:ab,ti OR cholecalciferol:ab,ti OR ' vitamin d '/exp

#2 intensive care unit:ab,ti OR ' intensive care unit '/exp OR intensive care:ab,ti OR ' intensive care '/exp OR critical care:ab,ti OR icu:ab,ti

#3 randomized controlled trial:ab,ti OR ' randomized controlled trial '/exp

#1 AND #2 AND #3

Scopus

#1 (TITLE-ABS-KEY (vitamin d) OR TITLE-ABS-KEY (cholecalciferol) OR TITLE-ABS-KEY (calcitriol) OR TITLE-ABS-KEY (hydroxyvitamin d) OR TITLE-ABS-KEY (dihydroxyvitamin d))

#2 (TITLE-ABS-KEY (intensive care) OR TITLE-ABS-KEY (critical care) OR TITLE-ABS-KEY (icu) OR TITLE-ABS-KEY (critical illness) OR TITLE-ABS-KEY (critically ill))

#3 (TITLE-ABS-KEY (randomized controlled trial)

#1 AND #2 AND #3
