# Supplementary material for: The Effect of Vitamin D Supplementation on Clinical Outcomes for Critically Ill Patients: A Systemic Review and Meta-Analysis of Randomized Clinical Trials
Source: Front Nutr. 2021 May 4;8:664940. doi: 10.3389/fnut.2021.664940 (PMC8129506; doi:10.3389/fnut.2021.664940)
Supplement: Supplementary file 3 [file Data_Sheet_3.PDF]

Additional file 3: Detailed information of included studies

**Title**	Short-Term Effects of Alfacalcidol on Hospital Length of Stay in Patients Undergoing Valve Replacement Surgery: A Randomized Clinical Trial
**First author (Year)**	Sandra N. Naguib 2020
**Study type**	Single-center, open-label, placebo-controlled trial
**Participants** (Intervention/Control)	
**Number**	45/41
**Male%**	37/53
**Age**	44±11/43±11
**Inclusion criteria**	The study population included adult patients (aged 18-65 years) scheduled to undergo elective mechanical valve replacement surgery
**Exclusion criteria**	Mixed valve replacement surgery with coronary artery bypass graft surgery, valvular replacement redo, and valve replacement surgery secondary to infective endocarditis, dialysis, alanine aminotransferase level 2 to 3 times the upper limit of normal, coexisting coronary heart disease, indication for vitamin D supplementation within the prior month, hypercalcemia (total calcium of >10.4 mg/dL), hyperphosphatemia (serum phosphate >4.5 mg/dL), and duration on mechanical ventilation of longer than 2 days
**Risk of Bias**	**Random sequence generation:** Low (table of random numbers)<br>**Allocation concealment:** Low (sealed envelope technique)<br>**Blinding of participants and personnel:** High (open-label)<br>**Blinding of outcome assessment:** Low<br>**Incomplete outcome data:** Low<br>**Selective reporting:** Low<br>**Other bias:** Low

**Title**	Neuroprotective Role of Oral Vitamin D Supplementation on Consciousness and Infammatory Biomarkers in Determining Severity Outcome in Acute Traumatic Brain Injury Patients: A Double-Blind Randomized Clinical Trial
**First author (Year)**	Swapnil Sharma 2020
**Study type**	Single-center, double-blind, placebo-controlled trial
**Participants** (Intervention/Control)	
**Number**	20/15
**Male%**	71.4 (Total)
**Age**	36.4 (Total)
**Inclusion criteria**	The inclusion criteria of this study were a Glasgow Coma Scale (GCS) score of 4–12, started on enteral nutrition within 24 h after admission and on mechanical ventilation in the ICU
**Exclusion criteria**	Patients with GCS > 12 or < 4; internal organ bleeding; limb fractures; history of underlying neurologic, metabolic, or psychiatric disorders; and alcohol or drug abuse
**Risk of Bias**	**Random sequence generation:** Low (computer-generated balanced block randomization)<br>**Allocation concealment:** Low (sealed envelope technique)<br>**Blinding of participants and personnel:** Low (double-blind, placebo-controlled)<br>**Blinding of outcome assessment:** Low<br>**Incomplete outcome data:** Low<br>**Selective reporting:** Unclear (inadequate information)<br>**Other bias:** Low

**Title**	Effect of Intravenous 25OHD Supplementation on Bone Turnover and Inflammation in Prolonged Critically Ill Patients
**First author (Year)**	Catherine Ingels 2020
**Study type**	Single-center, double-blind, placebo-controlled trial
**Participants** (Intervention/Control)	
**Number**	11/13
**Male%**	92/72
**Age**	58±10/52±18
**Inclusion criteria**	Patients with an anticipated stay in the intensive care unit (ICU) of more than 10 days
**Exclusion criteria**	Patients younger than 18 years, those suffering from chronic bone or kidney disease, and those who were treated with glucocorticoids prior to ICU admission
**Risk of Bias**	**Random sequence generation:** Unclear (inadequate information)<br>**Allocation concealment:** Unclear (inadequate information)<br>**Blinding of participants and personnel:** Low (double-blind, placebo-controlled)<br>**Blinding of outcome assessment:** Unclear (inadequate information)<br>**Incomplete outcome data:** Low<br>**Selective reporting:** Low (ISRCTN24385496)<br>**Other bias:** Low

**Title**	Early High-Dose Vitamin D3 for Critically Ill, Vitamin D–Deficient Patients
**First author (Year)**	Ginde 2019
**Study type**	Multicenter, double-blind, placebo-controlled trial
**Participants** (Intervention/Control)	
**Number**	538/540
**Male%**	57.4/55.9
**Age**	56.5±15.9/54.6±16.7
**Inclusion criteria**	1.Age ≥ 18 years<br>2.Intention to admit to ICU from emergency department, hospital ward, operating room, or outside facility<br>3.One or more of the following acute risk factors for ARDS and mortality contributing directly to the need for ICU admission: Pulmonary; Pneumonia; Aspiration; Smoke; Inhalation; Lung contusion; Mechanical ventilation for acute hypoxemic or hypercarbic respiratory failure Extra-Pulmonary; Shock; Sepsis; Pancreatitis<br>4.Vitamin D deficiency (screening 25OHD level <20 ng/mL)
**Exclusion criteria**	1.Inability to obtain informed consent<br>2.Unable to randomize within 12 hours of ICU admission decision<br>3.Unable to take study medication by mouth or enteral tube<br>4.Baseline serum calcium >10.2 mg/dL (2.54 mmol/L) or ionized calcium >5.2 mg/dL (1.30 mmol/L)<br>5.Known kidney stone in past year or history of multiple (>1) prior kidney stone episodes<br>6.Decision to withhold or withdraw life-sustaining treatment (patients are still eligible if they are committed to full support except cardiopulmonary resuscitation if a cardiac arrest occurs)<br>7.Expect < 48 hours survival<br>8.If no other risk factors present, a) mechanical ventilation primarily for airway protection, pain/agitation control, or procedure; or b) elective surgical patients with routine postoperative mechanical ventilation; or c) anticipated mechanical ventilation duration <24 hours; or d) chronic/home mechanical ventilation for chronic lung or neuromuscular disease (non-invasive ventilation used solely for sleep-disordered breathing is not an exclusion).<br>9.Prisoner<br>10.Pregnancy
**Risk of Bias**	**Random sequence generation:** Low (central electronic system and permuted blocks to randomly assign eligible patients)<br>**Allocation concealment:** Unclear (inadequate information)<br>**Blinding of participants and personnel:** Low (double-blind, placebo-controlled)<br>**Blinding of outcome assessment:** Low<br>**Incomplete outcome data:** Low<br>**Selective reporting:** Low (NCT03096314)<br>**Other bias:** Low

**Title**	Effect of High-Dose Vitamin D on Duration of Mechanical Ventilation in ICU Patients
**First author (Year)**	Miri 2019
**Study type**	Single-center, double-blind, placebo-controlled trial
**Participants** (Intervention/Control)	
**Number**	22/18
**Male%**	63.6/72.2
**Age**	52.0±22.1/56.0±22.1
**Inclusion criteria**	Adult (age between 18 and 65 years) mechanically ventilated patients.
**Exclusion criteria**	Refusal of the legal guardian of the patient to participate in the study, the patient's death in less than 72 h after enrolling in the study, renal dysfunction (GFR <30 mL/min), and the onset of dialysis during the study, vitamin D supplementation in the last 15 days, hypo/hyper-calcemia (adjusted calcium less than 8 mg/dL or above 10), liver failure (Child-Pough stage C), parathyroid dysfunction, CPIS (Clinical Pulmonary Infection Score) >6, INR > 1.5, platelet < 80,000 and hemodynamic disturbances (MAP less than 60 mmHg in three consecutive hours)
**Risk of Bias**	**Random sequence generation:** Low (permuted block randomization)<br>**Allocation concealment:** Unclear (inadequate information)<br>**Blinding of participants and personnel:** Low (double-blind, placebo-controlled)<br>**Blinding of outcome assessment:** Unclear (inadequate information)<br>**Incomplete outcome data:** Low<br>**Selective reporting:** Unclear (inadequate information)<br>**Other bias:** High (the condition of vitamin D deficiency in intervention group did not get significant improvement after 7-days intervention)

**Title**	The effect of supplementation of vitamin D in neurocritical care patients: Randomized Clinical Trial of Hypovitaminosis D (RECTIFY)
**First author (Year)**	Karsy 2019
**Study type**	Single-center, double-blind, placebo-controlled trial
**Participants** (Intervention/Control)	
**Number**	22/18
**Male%**	63.6/72.2
**Age**	52.0±22.1/56.0±22.1
**Inclusion criteria**	1.Patients >18 years of age<br>2.Patients admitted to the neurosurgery or neurology services<br>3.Patients admitted to a critical care unit<br>4.Informed consent<br>5.Expected to stay in the ICU for 48 hours or more<br>6.Vitamin D deficiency (<20ng/mL)
**Exclusion criteria**	1.Patients where a vitamin D level was not drawn within 48 hours of admission<br>2.Patients not randomized within 48 hours of admission<br>3.Readmitted patients to the critical care unit<br>4.Lack of informed consent<br>5.Prior supplementation with vitamin D<br>6.Severely impaired gastrointestinal function<br>7.Other trial participation<br>8.Pregnant or lactating women<br>9.Hypercalcemia (total calcium of >10.6 mg/dL or ionized serum calcium of >5.4 mg/dL<br>10.Tuberculosis history or clinical exam<br>11.Sarcoidosis history or clinical exam<br>12.Nephrolithiasis within the prior year<br>13.Patients not deemed suitable for study participation (ie, psychiatric disease, living remotely from the clinic, or prisoner status)<br>14.Pregnant or nursing women
**Risk of Bias**	**Random sequence generation:** Low (random number generator)<br>**Allocation concealment:** Low (managed by investigational drug pharmacy)<br>**Blinding of participants and personnel:** Low (double-blind, placebo-controlled)<br>**Blinding of outcome assessment:** Low<br>**Incomplete outcome data:** Low<br>**Selective reporting:** Low (NCT02881957)<br>**Other bias:** Low

**Title**	Effect of Oral Versus Intramuscular Vitamin D Replacement on Oxidative Stress and Outcomes in Traumatic Mechanical Ventilated Patients Admitted to Intensive Care Unit
**First author (Year)**	Hasanloei 2019
**Study type**	Single-center, no-blind, trial
**Participants** (Intervention/Control)	
**Number**	24 (Oral VD Group)/24 (Injection VD Group)/24 (Placebo Group)
**Male%**	70.8/45.8/50.0
**Age**	50.0±16.5/44.4±15.4/48.7±7.9
**Inclusion criteria**	Adult patients (age: 18–65 years) with an expected need of mechanical ventilation for at least 48 hours and at least 7 days' stay in the ICU, patients with a 25(OH)D serum level between 10 and 30 ng/mL, no contraindication for enteral feeding, no acute or chronic renal failure, and a Glasgow Coma Scale (GCS) score⩾9
**Exclusion criteria**	Failure of enteral feeding (complications such as such as aspiration, tube malpositioning or dislodgment, refeeding syndrome, medicationrelated complications, fluid imbalance, insertion-site infection, and agitation); receiving immunosuppressive drugs (cyclosporine and tacrolimus in any dose) 1 week before enrollment; and having immunosuppressive diseases, mechanical ventilation for >72 hours before entering the study, and a GCS score of <9
**Risk of Bias**	**Random sequence generation:** Unclear (inadequate information)<br>**Allocation concealment:** Unclear (inadequate information)<br>**Blinding of participants and personnel:** High (no-blind)<br>**Blinding of outcome assessment:** Unclear (inadequate information)<br>**Incomplete outcome data:** Low<br>**Selective reporting:** Unclear (inadequate information)<br>**Other bias:** Low

**Title**	Vitamin D to Prevent Lung Injury Following Esophagectomy—A Randomized, Placebo-Controlled Trial
**First author (Year)**	Parekh 2018
**Study type**	Single-center, double-blind, placebo-controlled trial
**Participants** (Intervention/Control)	
**Number**	40/39
**Male%**	90/79
**Age**	65/66
**Inclusion criteria**	Patients undergoing a planned thoracic esophagectomy and 18 years old or older if male, aged 55 or more than 2 years since menopause if female and were able to give written informed consent
**Exclusion criteria**	Patients with a known intolerance of VD, pregnant or breast feeding, sarcoidosis, tuberculosis, lymphoma, hyperparathyroidism, or nephrolithiasis, baseline serum adjusted calcium >2.65mmol/L, undergoing hemodialysis, or had a known diagnosis of chronic obstructive pulmonary disease with FEV1 less than 50% predicted or resting oxygen saturations less than 92% were ineligible
**Risk of Bias**	**Random sequence generation:** Low (computer-generated randomization sequence)<br>**Allocation concealment:** Unclear (inadequate information)<br>**Blinding of participants and personnel:** Low (double-blind, placebo-controlled)<br>**Blinding of outcome assessment:** Low<br>**Incomplete outcome data:** Low<br>**Selective reporting:** Unclear (inadequate information)<br>**Other bias:** High (Patients received intervention before entering ICU)

**Title**	Effect of Vitamin D Supplementation on Procalcitonin as Prognostic Biomarker in Patients with Ventilator Associated Pneumonia Complicated with Vitamin D Defciency
**First author (Year)**	Miroliaee 2017
**Study type**	Single-center, double-blind, placebo-controlled trial
**Participants** (Intervention/Control)	
**Number**	24 (Oral VD Group)/24 (Injection VD Group)/24 (Placebo Group)
**Male%**	70.8/45.8/50.0
**Age**	50.0±16.5/44.4±15.4/48.7±7.9
**Inclusion criteria**	Adult patients >18 years old who had been diagnosed with VAP
**Exclusion criteria**	Subjects with chronic renal failure (GFR<30cc/min), pancreatitis, hepatic insuffciency<br>with stage B or C of child-Pugh score, those with a history of cancer in the last 3 month or subjects undergoing chemotherapy, immune compromised patients, patients with VAP but with normal vitamin D levels (≥ 30 ng/mL) and those with coagulopathy (INR>1.5 or PTT>2 times of normal range or plt<100000/mL) were excluded
**Risk of Bias**	**Random sequence generation:** Unclear (inadequate information)<br>**Allocation concealment:** Unclear (inadequate information)<br>**Blinding of participants and personnel:** Low (double-blind)<br>**Blinding of outcome assessment:** Unclear (inadequate information)<br>**Incomplete outcome data:** Low<br>**Selective reporting:** Low (IRCT2014112920134N1)<br>**Other bias:** Low

**Title**	High dose vitamin D administration in ventilated intensive care unit patients: A pilot double blind randomized controlled trial
**First author (Year)**	Han 2016
**Study type**	Single-center, double-blind, placebo-controlled trial
**Participants** (Intervention/Control)	
**Number**	9 (Low dose group)/11 (High dose group)/10 (Placebo group)
**Male%**	55.6/72.7/60.0
**Age**	56.4±15.4/68.1±18.6/64.8±17.5
**Inclusion criteria**	1.Receiving care in an ICU;<br>2.Age greater than 18 years;<br>3.Expected to require mechanical ventilation for at least 72 hours after study entry;<br>4.Expected to survive and remain in the ICU for at least 96 hours after study entry;<br>5.Enteral access in place to enable delivery of vitamin D3 or placebo and are deemed to be able to tolerate enteral drug administration
**Exclusion criteria**	1.Inability to obtain or declined informed consent from the subject and/or legally authorized representative;<br>2.Current pregnancy;<br>3.Ongoing shock, [defined as unstable blood pressure despite vasopressor support and mean arterial pressure (MAP) < 60 mm Hg on at least 3 consecutive readings within a 3-hour period prior to study entry]; 4) current hypercalcemia (albumin-corrected serum calcium > 10.8 mg/dL or ionized calcium > 5.2 mg/dL);<br>5.History of therapy with highdose vitamin D3 (greater than or equal to 50,000 IU a week) to treat vitamin D deficiency, within previous 6 months;<br>6.History of disorders associated with hypercalcemia (history of cancer with history of hypercalcemia within the past 1 year, hyperparathyroidism, sarcoidosis, nephrolithiasis);<br>7.Chronic dialysis;<br>8.Known history of cirrhosis;<br>9.Known HIV;<br>10.Received any investigational drug within 60 days prior to study entry
**Risk of Bias**	**Random sequence generation:** Unclear (inadequate information)<br>**Allocation concealment:** Unclear (inadequate information)<br>**Blinding of participants and personnel:** Low (double-blind)<br>**Blinding of outcome assessment:** Low<br>**Incomplete outcome data:** Low<br>**Selective reporting:** Low<br>**Other bias:** Low

**Title**	Effect of Cholecalciferol Supplementation on Vitamin D Status and Cathelicidin Levels in Sepsis: A Randomized, Placebo-Controlled Trial
**First author (Year)**	Quraishi 2015
**Study type**	Single-center, double-blind, placebo-controlled trial
**Participants** (Intervention/Control)	
**Number**	10 (Low dose group)/10 (High dose group)/10 (Placebo group)
**Male%**	60/60/60
**Age**	64(55-66)/62(59-67)/65(58-70)
**Inclusion criteria**	1.Receiving care in an ICU;<br>2.Age greater than 18 years;<br>3.Expected to require mechanical ventilation for at least 72 hours after study entry;<br>4.Expected to survive and remain in the ICU for at least 96 hours after study entry;<br>5.Enteral access in place to enable delivery of vitamin D3 or placebo and are deemed to be able to tolerate enteral drug administration
**Exclusion criteria**	All adult patients, at least 18 years old, admitted to the medical or surgical ICUs at MGH, and within 24 hours of new-onset sepsis
**Risk of Bias**	**Random sequence generation:** Low (computer-generated block randomization algorithm)<br>**Allocation concealment:** Low (managed by research pharmacy)<br>**Blinding of participants and personnel:** Low (double-blind)<br>**Blinding of outcome assessment:** Low<br>**Incomplete outcome data:** Low<br>**Selective reporting:** Low (NCT01896544)<br>**Other bias:** Low

**Title**	Effect of High-Dose Vitamin D3 on Hospital Length of Stay in Critically Ill Patients With Vitamin D Deficiency<br>The VITdAL-ICU Randomized Clinical Trial
**First author (Year)**	Amrein 2014
**Study type**	Single-center, double-blind, placebo-controlled trial
**Participants** (Intervention/Control)	
**Number**	237/238
**Male%**	65.0/65.1
**Age**	63.9±15.5/65.3±14.0
**Inclusion criteria**	Patients who were 18 years or older, expected to stay in the ICU for 48 hours or more, and found to have a 25-hydroxyvitamin D level of 20 ng/mL (to convert to nmol/L, multiply by 2.496) or lower were eligible for study participation
**Exclusion criteria**	Patients who met any of the following criteria were not eligible to participate in the trial: severely impaired gastrointestinal function; other trial participation, including previous participation in the pilot trial; pregnant or lactating women; hypercalcemia (total calcium of >10.6 mg/dL or ionized serum calcium of >5.4 mg/dL [to convert both to mmol/L, multiply by 0.25]); tuberculosis; sarcoidosis; nephrolithiasis within the prior year; and patients not deemed suitable for study participation (ie, psychiatric disease, living remotely from the clinic, or prisoner status)
**Risk of Bias**	**Random sequence generation:** Low (randomizer for Clinical Trials tool developed at the Medical University of Graz)<br>**Allocation concealment:** Low<br>**Blinding of participants and personnel:** Low (double-blind)<br>**Blinding of outcome assessment:** Low<br>**Incomplete outcome data:** Low<br>**Selective reporting:** Low (NCT01130181)<br>**Other bias:** Low

**Title**	Randomized Controlled Trial of Calcitriol in Severe Sepsis
**First author (Year)**	Leaf 2014
**Study type**	Single-center, double-blind, placebo-controlled trial
**Participants** (Intervention/Control)	
**Number**	36/31
**Male%**	61/48
**Age**	68 (54-70)/58 (49-69)
**Inclusion criteria**	Inclusion criteria were age greater than or equal to 18 years, severe sepsis or septic shock, and presence of an arterial or central venous catheter (for blood drawing)
**Exclusion criteria**	Exclusion criteria were serum calcium greater than or equal to 10.0 mg/dl or phosphate greater than or equal to 6.0 mg/dl within the previous 48 hours; current or recent therapy (within the previous 7 d) with nutritional vitamin D at doses greater than 1,000 IU per day or 1,25D at any dose; history of primary parathyroid disease, metabolic bone disease, sarcoidosis, or end-stage renal disease; AKI receiving intermittent RRT (patients receiving continuous RRT were eligible); expected to die or leave the ICU within 48 hours; and pregnancy
**Risk of Bias**	**Random sequence generation:** Low (computer-generated assignment sequence)<br>**Allocation concealment:** Unclear (inadequate information)<br>**Blinding of participants and personnel:** Low (physicians, nurses, patients, and study<br>investigators were masked to treatment assignment)<br>**Blinding of outcome assessment:** Low<br>**Incomplete outcome data:** Low<br>**Selective reporting:** Low (NCT01689441)<br>**Other bias:** Low

**Title**	Short-term effects of high-dose oral vitamin D3 in critically ill vitamin D deficient patients: a randomized, double-blind, placebo-controlled pilot study
**First author (Year)**	Amrein 2011
**Study type**	Single-center, double-blind, placebo-controlled trial
**Participants** (Intervention/Control)	
**Number**	12/13
**Male%**	77/75
**Age**	64.1±16.4/61.1±16.7
**Inclusion criteria**	25(OH)D-deficient adult patients (levels ≤20 ng/ml) with an expected stay in the medical ICU of more than 48 hours
**Exclusion criteria**	Exclusion criteria included moribund patients expected to die within 24 hours, hypercalcemia, ileus, pregnancy, and a history of kidney stones, sarcoidosis or tuberculosis
**Risk of Bias**	**Random sequence generation:** Unclear (inadequate information)<br>**Allocation concealment:** Low (randomization was performed with sealed envelopes)<br>**Blinding of participants and personnel:** Low (all aspects of the trial were performed in a double-blind fashion)<br>**Blinding of outcome assessment:** Low<br>**Incomplete outcome data:** Low<br>**Selective reporting:** Unclear (inadequate information)<br>**Other bias:** Low
