# Supplementary material for: The Effect of Vitamin D Supplementation on Clinical Outcomes for Critically Ill Patients: A Systemic Review and Meta-Analysis of Randomized Clinical Trials
Source: Front Nutr. 2021 May 4;8:664940. doi: 10.3389/fnut.2021.664940 (PMC8129506; doi:10.3389/fnut.2021.664940)
Supplement: Supplementary file 4 [file Data_Sheet_4.PDF]

Additional file 4: Excluded studies with reasons

Study	Reason for exclusion
Ved 2020[1]	Prospective observational cohort study
Martucci 2019[2]	Secondary analysis of the VITdAL-ICU study
Langlois 2019[3]	Review article, individual studies were reviewed for eligibility
Otero 2019[4]	Retrospective cohort study
Viglianti 2019[5]	Secondary analysis of the VITdAL-ICU study
Amrein 2019[6]	Study protocol
Friedberg 2019[7]	Patients not considered to be adult critically ill patients
Zheng 2018[8]	No concerned outcomes
Slow 2018[9]	Patients not considered to be adult critically ill patients
Langlois 2018[10]	Meta-analysis, individual studies were reviewed for eligibility
Lee[11] 2018	Patients not considered to be adult critically ill patients
Bi 2018[12]	Meta-analysis, individual studies were reviewed for eligibility
Smith 2018[13]	No concerned outcomes
Han 2018[14]	Used the same patient cohort with previous study
Schwetz 2017[15]	Secondary analysis of the VITdAL-ICU study
Otero 2017[16]	Retrospective study
Han 2017[17]	Used the same patient cohort with previous study
Weng 2017[18]	Meta-analysis, individual studies were reviewed for eligibility
Putzu 2017[19]	Meta-analysis, individual studies were reviewed for eligibility
Amrein 2016[20]	Secondary analysis of the VITdAL-ICU study
Alizadeh 2016[21]	No concerned outcomes
Quraishi 2016[22]	Secondary analysis of an observational cohort study
Nair 2015[23]	No placebo group
Quraishi 2014[24]	Inappropriate Intervention
Via 2012[25]	Inappropriate Intervention
Grossmann 2012[26]	Patients not considered to be adult critically ill patients
Venkatram 2011[27]	Retrospective study
Van 2003[28]	No placebo group

Reference:

1. Ved R, Taylor P, Stewart P, et al. Vitamin D deficiency is endemic in neurosurgical patients and is associated with a longer length of inpatient stay. *Endocrinology, diabetes & metabolism* 2020;3(1):e00097. doi: 10.1002/edm2.97 [published Online First: 2020/01/11]
2. Martucci G, McNally D, Parekh D, et al. Trying to identify who may benefit most from future vitamin D intervention trials: a post hoc analysis from the VITDAL-ICU study excluding the early deaths. *Critical care (London, England)* 2019;23(1):200. doi: 10.1186/s13054-019-2472-z [published Online First: 2019/06/06]
3. Langlois PL, D'Aragon F, Manzanares W. Vitamin D in the ICU: More sun for critically ill adult patients? *Nutrition (Burbank, Los Angeles County, Calif)* 2019;61:173-78. doi: 10.1016/j.nut.2018.11.001 [published Online First: 2019/02/08]
4. Otero TMN, Canales C, Yeh DD, et al. Vitamin D Status Is Associated With Development of

Hospital-Acquired Pressure Injuries in Critically Ill Surgical Patients. *Nutrition in clinical practice : official publication of the American Society for Parenteral and Enteral Nutrition* 2019;34(1):142-47. doi: 10.1002/ncp.10184 [published Online First: 2018/08/14]

5. Viglianti E, Zajic P, Iwashyna T, et al. Neither vitamin D levels nor supplementation are associated with persistent critical illness. *Critical care medicine* 2019;47(1)

6. Amrein K, Parekh D, Westphal S, et al. Effect of high-dose vitamin D3 on 28-day mortality in adult critically ill patients with severe vitamin D deficiency: a study protocol of a multicentre, placebo-controlled double-blind phase III RCT (the VITDALIZE study). *BMJ open* 2019;9(11):e031083. doi: 10.1136/bmjopen-2019-031083 [published Online First: 2019/11/15]

7. Friedberg JW, Brady MT, Strawderman M, et al. Indolent lymphoma and Vitamin D (ILyAD): A double-blind, randomized, placebo-controlled trial to evaluate the supplemental effect of vitamin d on event-free survival in patients with low tumor burden indolent non-hodgkin lymphoma treated with rituximab. *Blood* 2019;134 doi: 10.1182/blood-2019-127320

8. Zheng CM, Wu CC, Hung CF, et al. Cholecalciferol Additively Reduces Serum Parathyroid Hormone Levels in Severe Secondary Hyperparathyroidism Treated with Calcitriol and Cinacalcet among Hemodialysis Patients. *Nutrients* 2018;10(2) doi: 10.3390/nu10020196 [published Online First: 2018/02/15]

9. Slow S, Epton M, Storer M, et al. Effect of adjunctive single high-dose vitamin D3 on outcome of community-acquired pneumonia in hospitalised adults: The VIDCAPS randomised controlled trial. *Scientific reports* 2018;8(1):13829. doi: 10.1038/s41598-018-32162-2 [published Online First: 2018/09/16]

10. Langlois PL, Szwec C, D'Aragon F, et al. Vitamin D supplementation in the critically ill: A systematic review and meta-analysis. *Clinical nutrition (Edinburgh, Scotland)* 2018;37(4):1238-46. doi: 10.1016/j.clnu.2017.05.006 [published Online First: 2017/05/28]

11. Lee DR, Bowden S, Shaikhkhalil A, et al. Effectiveness of single, high-dose vitamin D replacement therapy (stoss therapy) in patients with inflammatory bowel disease and hypovitaminosis. *Journal of Pediatric Gastroenterology and Nutrition* 2018;67:S339-S40. doi: 10.1097/MPG.0000000000002164

12. Bi WG, Nuyt AM, Weiler H, et al. Association Between Vitamin D Supplementation During Pregnancy and Offspring Growth, Morbidity, and Mortality: A Systematic Review and Meta-analysis. *JAMA pediatrics* 2018;172(7):635-45. doi: 10.1001/jamapediatrics.2018.0302 [published Online First: 2018/05/31]

13. Smith EM, Jones JL, Han JE, et al. High-Dose Vitamin D3 Administration Is Associated With Increases in Hemoglobin Concentrations in Mechanically Ventilated Critically Ill Adults: A Pilot Double-Blind, Randomized, Placebo-Controlled Trial. *JPEN Journal of parenteral and enteral nutrition* 2018;42(1):87-94. doi: 10.1177/0148607116678197 [published Online First: 2018/03/06]

14. Han JE, Alvarez JA, Staitieh B, et al. Oxidative stress in critically ill ventilated adults: effects of vitamin D3 and associations with alveolar macrophage function. *European journal of clinical nutrition* 2018;72(5):744-51. doi: 10.1038/s41430-017-0047-0 [published Online First: 2017/12/31]

15. Schwetz V, Schnedl C, Urbanic-Purkart T, et al. Effect of vitamin D3 on bone turnover markers in critical illness: post hoc analysis from the VITdAL-ICU study. *Osteoporosis international : a*

journal established as result of cooperation between the European Foundation for Osteoporosis and the National Osteoporosis Foundation of the USA 2017;28(12):3347-54. doi: 10.1007/s00198-017-4190-1 [published Online First: 2017/08/27]

16. Otero T, Canales C, Yeh DD, et al. Vitamin D status is associated with the development of hospital-acquired pressure ulcers in critically ill surgical patients. *Journal of Parenteral and Enteral Nutrition* 2017;41(2):289. doi: 10.1177/0148607116686023
17. Han JE, Alvarez JA, Jones JL, et al. Impact of high-dose vitamin D3 on plasma free 25-hydroxyvitamin D concentrations and antimicrobial peptides in critically ill mechanically ventilated adults. *Nutrition (Burbank, Los Angeles County, Calif)* 2017;38:102-08. doi: 10.1016/j.nut.2017.02.002 [published Online First: 2017/05/21]
18. Weng H, Li JG, Mao Z, et al. Randomised trials of vitamin D3 for critically ill patients in adults: systematic review and meta-analysis with trial sequential analysis. *Intensive care medicine* 2017;43(2):277-78. doi: 10.1007/s00134-016-4591-1 [published Online First: 2016/10/21]
19. Putzu A, Belletti A, Cassina T, et al. Vitamin D and outcomes in adult critically ill patients. A systematic review and meta-analysis of randomized trials. *Journal of critical care* 2017;38:109-14. doi: 10.1016/j.jcrc.2016.10.029 [published Online First: 2016/11/25]
20. Amrein K, McNally JD, Dobnig H, et al. High-dose cholecalciferol in critically ill patients with liver cirrhosis. *Journal of internal medicine* 2016;279(3):309-10. doi: 10.1111/joim.12457 [published Online First: 2015/12/29]
21. Alizadeh N, Khalili H, Mohammadi M, et al. Effect of vitamin D on stress-induced hyperglycaemia and insulin resistance in critically ill patients. *International journal of clinical practice* 2016;70(5):396-405. doi: 10.1111/ijcp.12795 [published Online First: 2016/04/20]
22. Quraishi SA, McCarthy C, Blum L, et al. Plasma 25-Hydroxyvitamin D Levels at Initiation of Care and Duration of Mechanical Ventilation in Critically Ill Surgical Patients. *JPEN Journal of parenteral and enteral nutrition* 2016;40(2):273-8. doi: 10.1177/0148607114566276 [published Online First: 2015/01/08]
23. Nair P, Venkatesh B, Lee P, et al. A Randomized Study of a Single Dose of Intramuscular Cholecalciferol in Critically Ill Adults. *Critical care medicine* 2015;43(11):2313-20. doi: 10.1097/ccm.0000000000001201 [published Online First: 2015/07/18]
24. Quraishi SA, Bittner EA, Blum L, et al. Prospective study of vitamin D status at initiation of care in critically ill surgical patients and risk of 90-day mortality. *Critical care medicine* 2014;42(6):1365-71. doi: 10.1097/ccm.0000000000000210 [published Online First: 2014/02/22]
25. Via MA, Potenza MV, Hollander J, et al. Intravenous ibandronate acutely reduces bone hyperresorption in chronic critical illness. *Journal of intensive care medicine* 2012;27(5):312-8. doi: 10.1177/0885066611402156 [published Online First: 2011/03/26]
26. Grossmann RE, Zughaier SM, Kumari M, et al. Pilot study of vitamin D supplementation in adults with cystic fibrosis pulmonary exacerbation: A randomized, controlled trial. *Dermato-endocrinology* 2012;4(2):191-7. doi: 10.4161/derm.20332 [published Online First: 2012/08/29]
27. Venkatram S, Chilimuri S, Adrish M, et al. Vitamin D deficiency is associated with mortality in the medical intensive care unit. *Critical care (London, England)* 2011;15(6):R292. doi: 10.1186/cc10585 [published Online First: 2011/12/14]
28. Van den Berghe G, Van Roosbroeck D, Vanhove P, et al. Bone turnover in prolonged critical illness:

effect of vitamin D. *The Journal of clinical endocrinology and metabolism* 2003;88(10):4623-32. doi: 10.1210/jc.2003-030358 [published Online First: 2003/10/15]
