# Supplementary material for: The Effect of Vitamin D Supplementation on Clinical Outcomes for Critically Ill Patients: A Systemic Review and Meta-Analysis of Randomized Clinical Trials
Source: Front Nutr. 2021 May 4;8:664940. doi: 10.3389/fnut.2021.664940 (PMC8129506; doi:10.3389/fnut.2021.664940)
Supplement: Supplementary file 5 [file Data_Sheet_5.PDF]

Additional files 5

e-Figure 1: Risk of bias assessment
e-Figure 2: Publication bias assessment by funnel plot and Egger's test
e-Figure 3: Effect of Vitamin D administration on long-term mortality in critically ill patients
e-Figure 4: Sensitivity analysis for primary outcome by omitting each one at a time
e-Figure 5: Sensitivity analysis for secondary outcomes by omitting each one at a time
e-Figure 6: Subgroup analysis for primary outcome
e-Figure 7: Subgroup analysis for length of ICU stay
e-Figure 8: Subgroup analysis for length of hospital stay
e-Figure 9: Subgroup analysis for duration of MV
e-Figure 10: Effect of Vitamin D administration on infection in critically ill patients

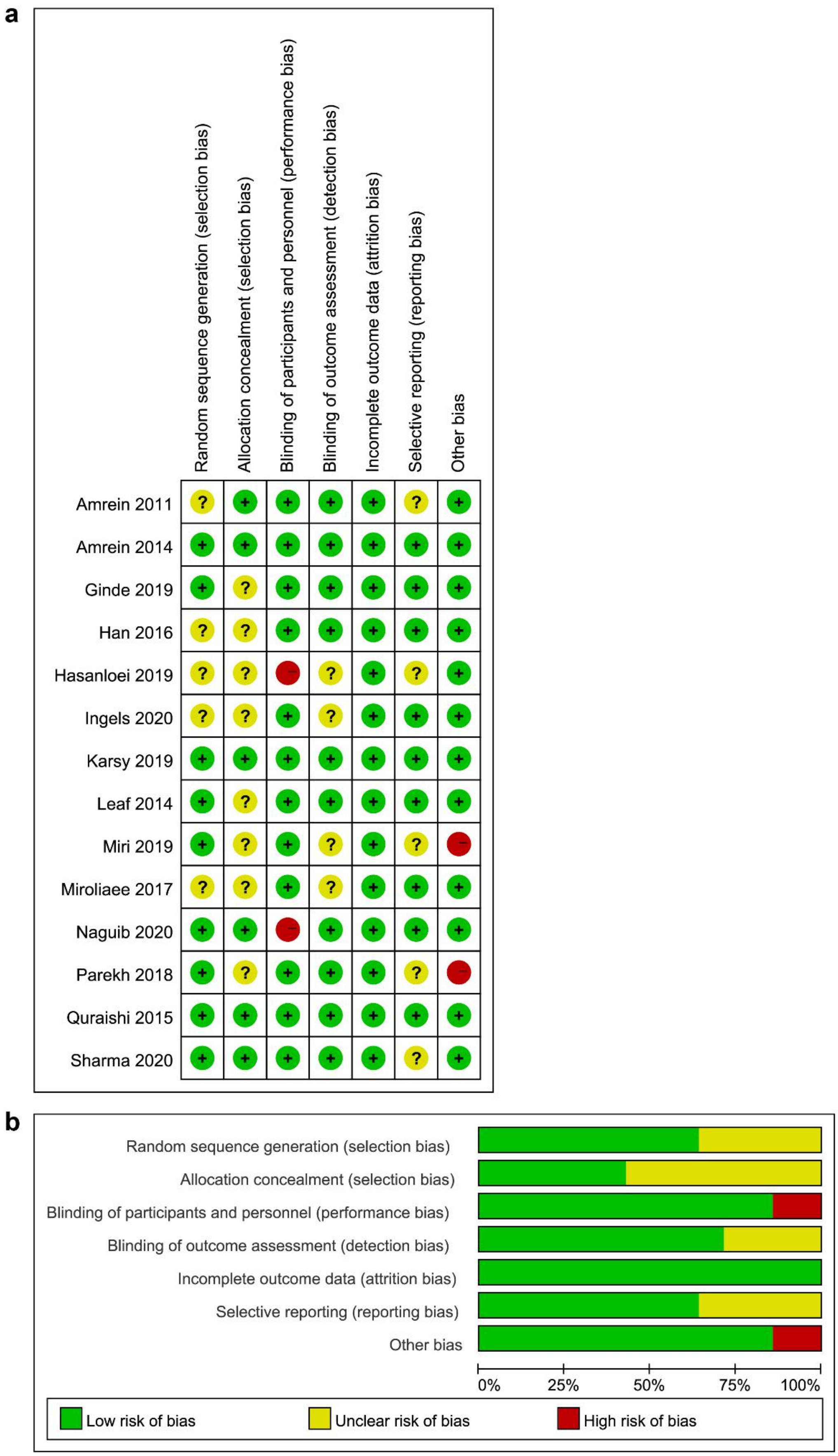

e-Figure 1: Risk of bias assessment. (a) risk of bias summary; (b) risk of bias graph.

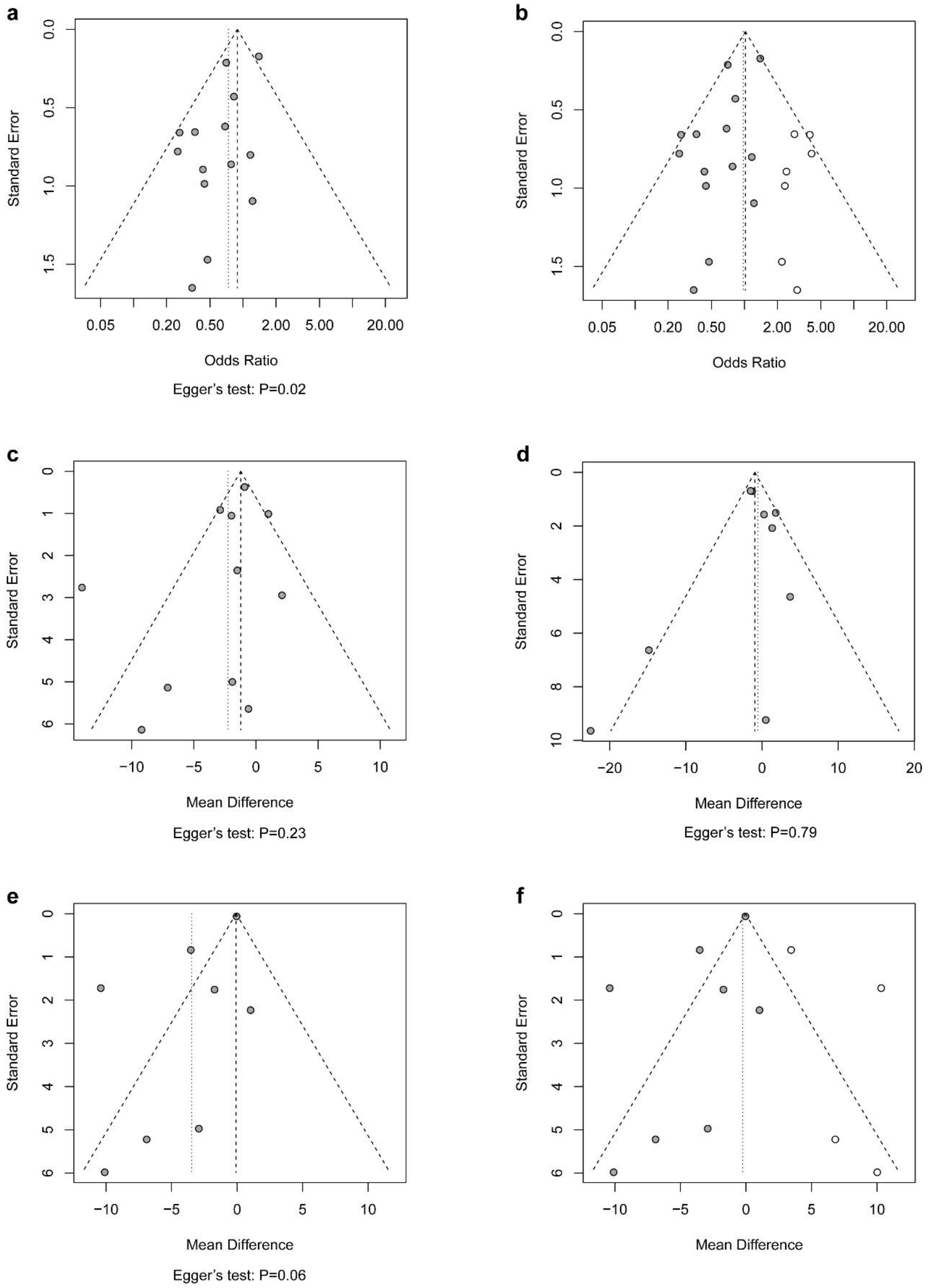

e-Figure 2: Publication bias assessment by funnel plot and Egger's test. (a) funnel plot for mortality; (b) symmetrical funnel plot after trim and fill method for mortality; (c) funnel plot for length of ICU stay; (d) funnel plot for length of hospital stay; (e) funnel plot for duration of MV; (b) symmetrical funnel plot after trim and fill method for duration of MV.

Study or Subgroup	Experimental Events	Experimental Total	Control Events	Control Total	Weight	Odds Ratio M-H, Random, 95% CI
Amrein 2014	83	237	102	238	41.7%	0.72 [0.50, 1.04]
Ginde 2019	123	531	109	528	52.9%	1.16 [0.87, 1.55]
Han 2016	5	20	2	10	2.9%	1.33 [0.21, 8.49]
Parekh 2018	2	33	2	35	2.5%	1.06 [0.14, 8.03]
**Total (95% CI)**		**821**		**811**	**100.0%**	**0.95 [0.69, 1.31]**
Total events	213		215			

Heterogeneity: Tau² = 0.03; Chi² = 4.07, df = 3 (P = 0.25); I² = 26%

Test for overall effect: Z = 0.31 (P = 0.76)

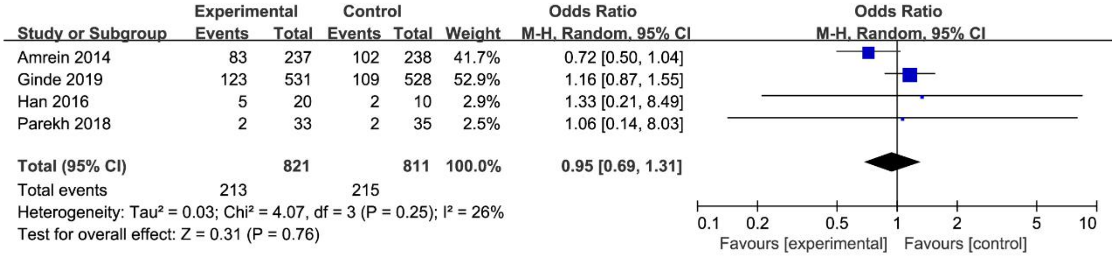

e-Figure 3: Effect of Vitamin D administration on long-term mortality in critically ill patients

Study	Odds Ratio MH, Random, 95% CI
Omitting Amrein 2011	0.71 [0.49; 1.02]
Omitting Amrein 2014	0.71 [0.47; 1.08]
Omitting Ginde 2019	0.63 [0.47; 0.85]
Omitting Han 2016	0.73 [0.51; 1.04]
Omitting Hasanloei 2019	0.79 [0.58; 1.10]
Omitting Ingels 2020	0.71 [0.50; 1.02]
Omitting Karsy 2019	0.70 [0.47; 1.03]
Omitting Leaf 2014	0.72 [0.50; 1.05]
Omitting Miri 2019	0.78 [0.55; 1.09]
Omitting Miroliaee 2017	0.82 [0.61; 1.12]
Omitting Naguib 2020	0.74 [0.52; 1.06]
Omitting Parekh 2018	0.73 [0.51; 1.04]
Omitting Quraishi 2015	0.72 [0.50; 1.03]
Omitting Sharma 2020	0.74 [0.52; 1.05]
**Total (95% CI)**	**0.73 [0.52; 1.03]**

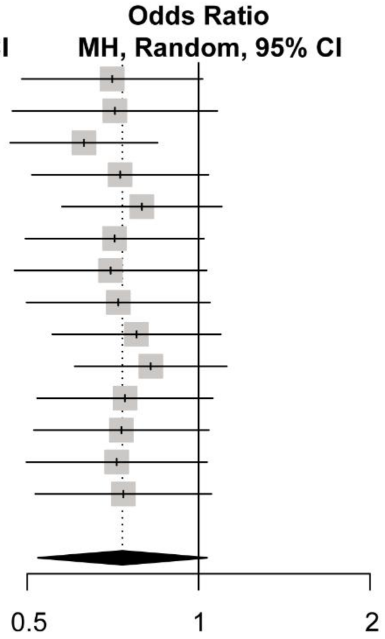

e-Figure 4: Sensitivity analysis for primary outcome by omitting each one at a time

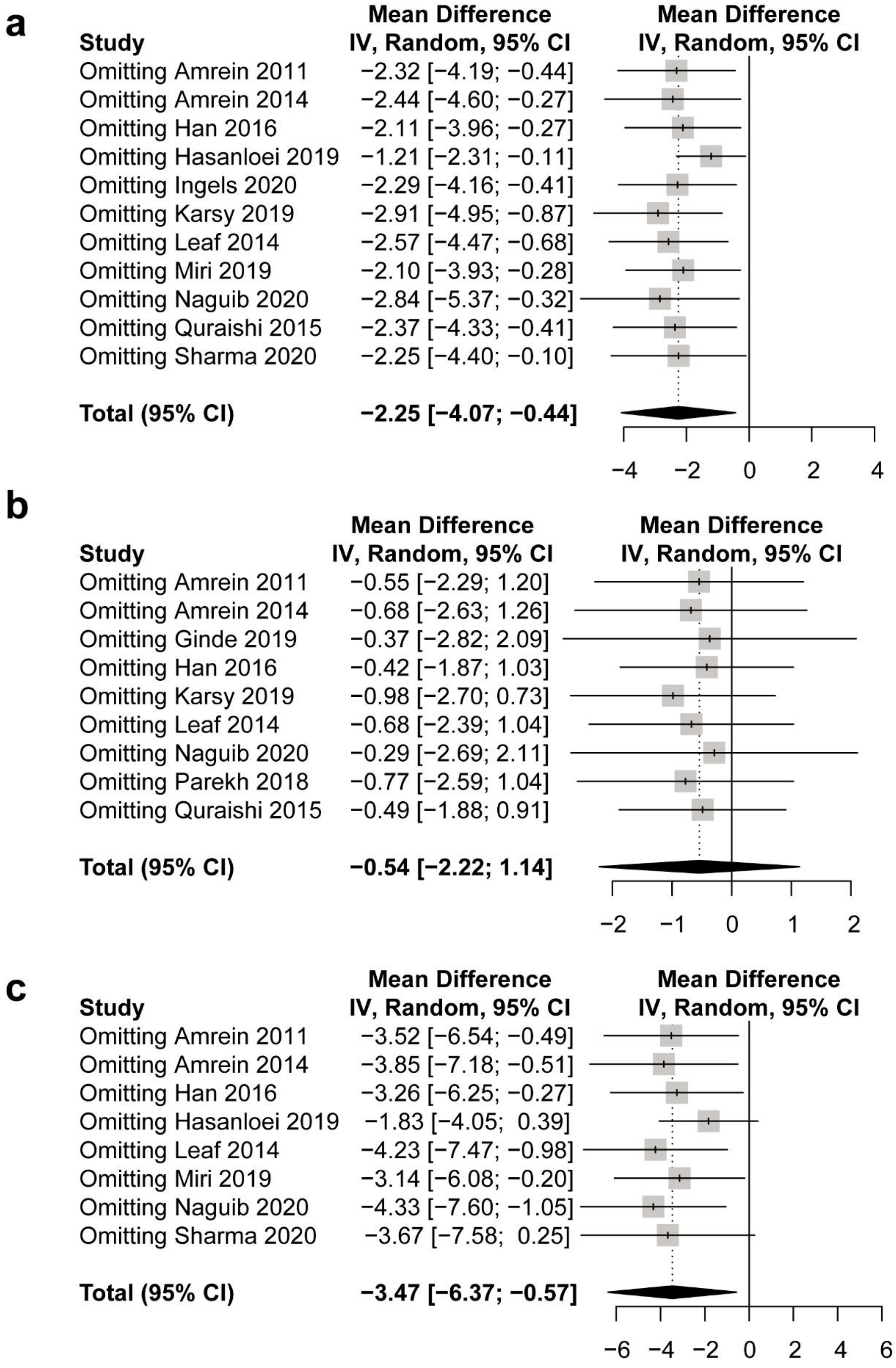

e-Figure 5: Sensitivity analysis for secondary outcomes by omitting each one at a time. (a) length of ICU stay; (b) length of hospital stay; (c) duration of MV

a

Study or Subgroup	Experimental Events	Experimental Total	Control Events	Control Total	Weight	Odds Ratio M-H, Random, 95% CI
**Low**						
Han 2016	1	20	1	10	1.0%	0.47 [0.03, 8.46]
Hasanloei 2019 Cohort A	2	24	5	24	2.6%	0.35 [0.06, 1.99]
Leaf 2014	6	36	7	31	5.1%	0.69 [0.20, 2.31]
Naguib 2020	2	45	4	41	2.6%	0.43 [0.07, 2.48]
Quraishi 2015 Cohort A	3	10	3	10	2.2%	1.00 [0.15, 6.77]
Sharma 2020	2	20	3	15	2.2%	0.44 [0.06, 3.07]
**Subtotal (95% CI)**		**155**		**131**	**15.8%**	**0.55 [0.27, 1.12]**
Total events	16		23			
Heterogeneity: Tau² = 0.00; Chi² = 0.91, df = 5 (P = 0.97); I² = 0%						
Test for overall effect: Z = 1.64 (P = 0.10)						
**High**						
Amrein 2011	6	12	6	13	3.2%	1.17 [0.24, 5.62]
Amrein 2014	52	237	68	238	23.3%	0.70 [0.46, 1.07]
Ginde 2019	92	531	69	528	27.7%	1.39 [0.99, 1.96]
Hasanloei 2019 Cohort B	1	24	5	24	1.7%	0.17 [0.02, 1.54]
Ingels 2020	2	11	2	13	1.8%	1.22 [0.14, 10.48]
Karsy 2019	11	134	13	133	9.5%	0.83 [0.36, 1.91]
Leaf 2014	6	36	7	31	5.1%	0.69 [0.20, 2.31]
Miri 2019	8	22	11	18	4.6%	0.36 [0.10, 1.32]
Miroliaee 2017	5	24	11	22	4.6%	0.26 [0.07, 0.96]
Parekh 2018	0	33	1	35	0.8%	0.34 [0.01, 8.73]
Quraishi 2015 Cohort B	2	10	3	10	1.9%	0.58 [0.07, 4.56]
**Subtotal (95% CI)**		**1074**		**1065**	**84.2%**	**0.75 [0.51, 1.11]**
Total events	185		196			
Heterogeneity: Tau² = 0.13; Chi² = 16.28, df = 10 (P = 0.09); I² = 39%						
Test for overall effect: Z = 1.44 (P = 0.15)						
**Total (95% CI)**		**1229**		**1196**	**100.0%**	**0.76 [0.57, 1.02]**
Total events	201		219			
Heterogeneity: Tau² = 0.05; Chi² = 19.00, df = 16 (P = 0.27); I² = 16%						
Test for overall effect: Z = 1.81 (P = 0.07)						
Test for subgroup differences: Chi² = 0.55, df = 1 (P = 0.46), I² = 0%						

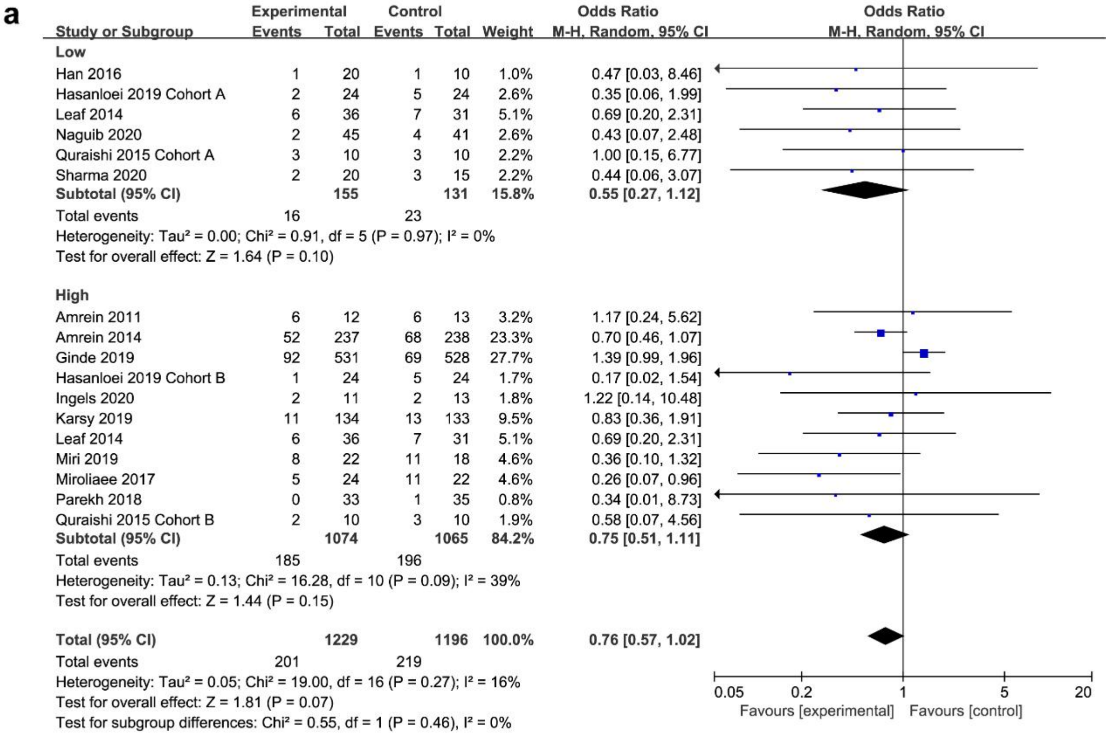

b

Study or Subgroup	Experimental Events	Experimental Total	Control Events	Control Total	Weight	Odds Ratio M-H, Random, 95% CI
**Severe**						
Amrein 2014 Cohort A	20	98	37	102	13.7%	0.45 [0.24, 0.85]
Ginde 2019	92	531	69	528	20.2%	1.39 [0.99, 1.96]
Ingels 2020	2	11	2	13	2.4%	1.22 [0.14, 10.48]
Miri 2019	8	22	11	18	5.7%	0.36 [0.10, 1.32]
**Subtotal (95% CI)**		**662**		**661**	**41.9%**	**0.73 [0.32, 1.68]**
Total events	122		119			
Heterogeneity: Tau² = 0.46; Chi² = 12.12, df = 3 (P = 0.007); I² = 75%						
Test for overall effect: Z = 0.74 (P = 0.46)						
**Less-severe**						
Amrein 2011	6	12	6	13	4.1%	1.17 [0.24, 5.62]
Amrein 2014 Cohort B	32	139	31	136	15.1%	1.01 [0.58, 1.78]
Han 2016	1	20	1	10	1.4%	0.47 [0.03, 8.46]
Hasanloei 2019	3	48	5	24	4.3%	0.25 [0.05, 1.17]
Karsy 2019	11	134	13	133	10.2%	0.83 [0.36, 1.91]
Leaf 2014	6	36	7	31	6.2%	0.69 [0.20, 2.31]
Miroliaee 2017	5	24	11	22	5.6%	0.26 [0.07, 0.96]
Naguib 2020	2	45	4	41	3.4%	0.43 [0.07, 2.48]
Parekh 2018	0	33	1	35	1.1%	0.34 [0.01, 8.73]
Quraishi 2015	5	20	3	10	3.6%	0.78 [0.14, 4.21]
Sharma 2020	2	20	3	15	2.9%	0.44 [0.06, 3.07]
**Subtotal (95% CI)**		**531**		**470**	**58.1%**	**0.72 [0.50, 1.03]**
Total events	73		85			
Heterogeneity: Tau² = 0.00; Chi² = 6.87, df = 10 (P = 0.74); I² = 0%						
Test for overall effect: Z = 1.81 (P = 0.07)						
**Total (95% CI)**		**1193**		**1131**	**100.0%**	**0.71 [0.50, 1.00]**
Total events	195		204			
Heterogeneity: Tau² = 0.13; Chi² = 21.34, df = 14 (P = 0.09); I² = 34%						
Test for overall effect: Z = 1.95 (P = 0.05)						
Test for subgroup differences: Chi² = 0.00, df = 1 (P = 0.98), I² = 0%						

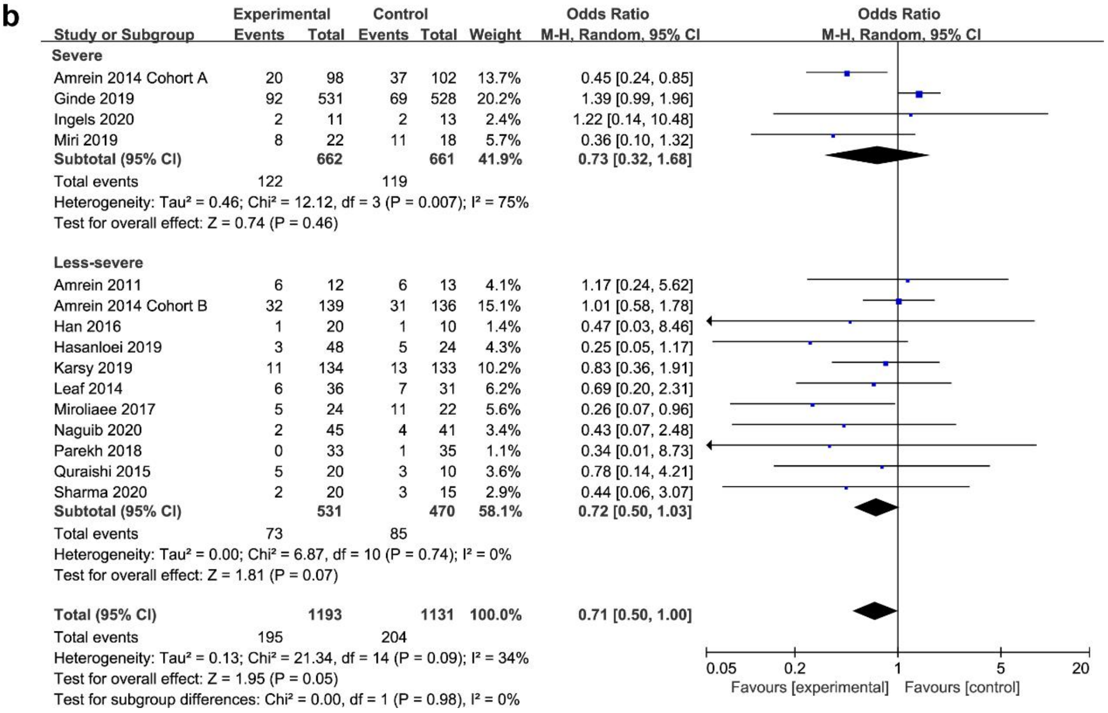

e-Figure 6: Subgroup analysis for primary outcome. (a) low dose of Vitamin D administration versus high; (b) severe Vitamin D deficiency versus less-severe

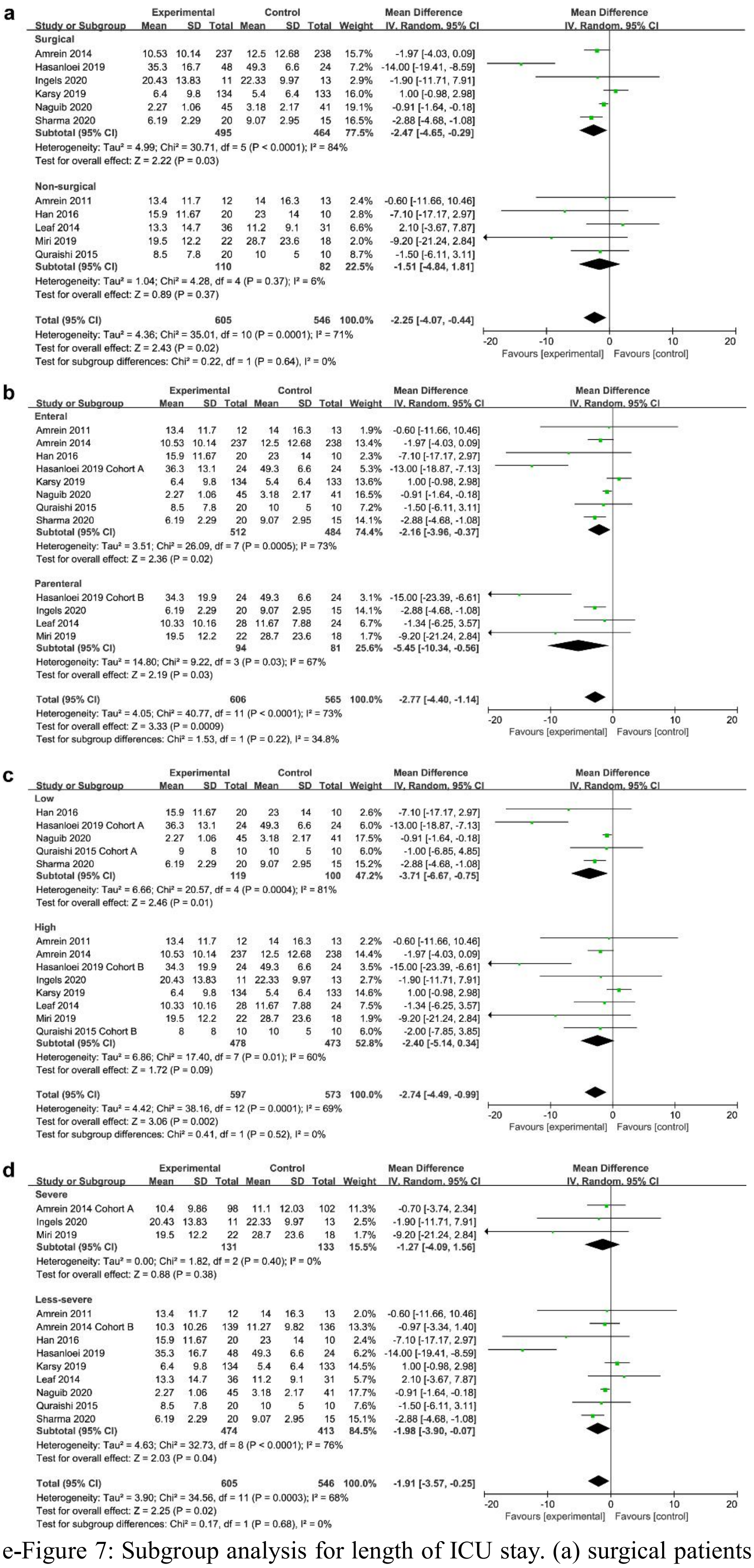

e-Figure 7: Subgroup analysis for length of ICU stay. (a) surgical patients versus non-surgical; (b) enteral route versus parenteral; (c) low dose of Vitamin D administration versus high; (d) severe Vitamin D deficiency versus less-severe

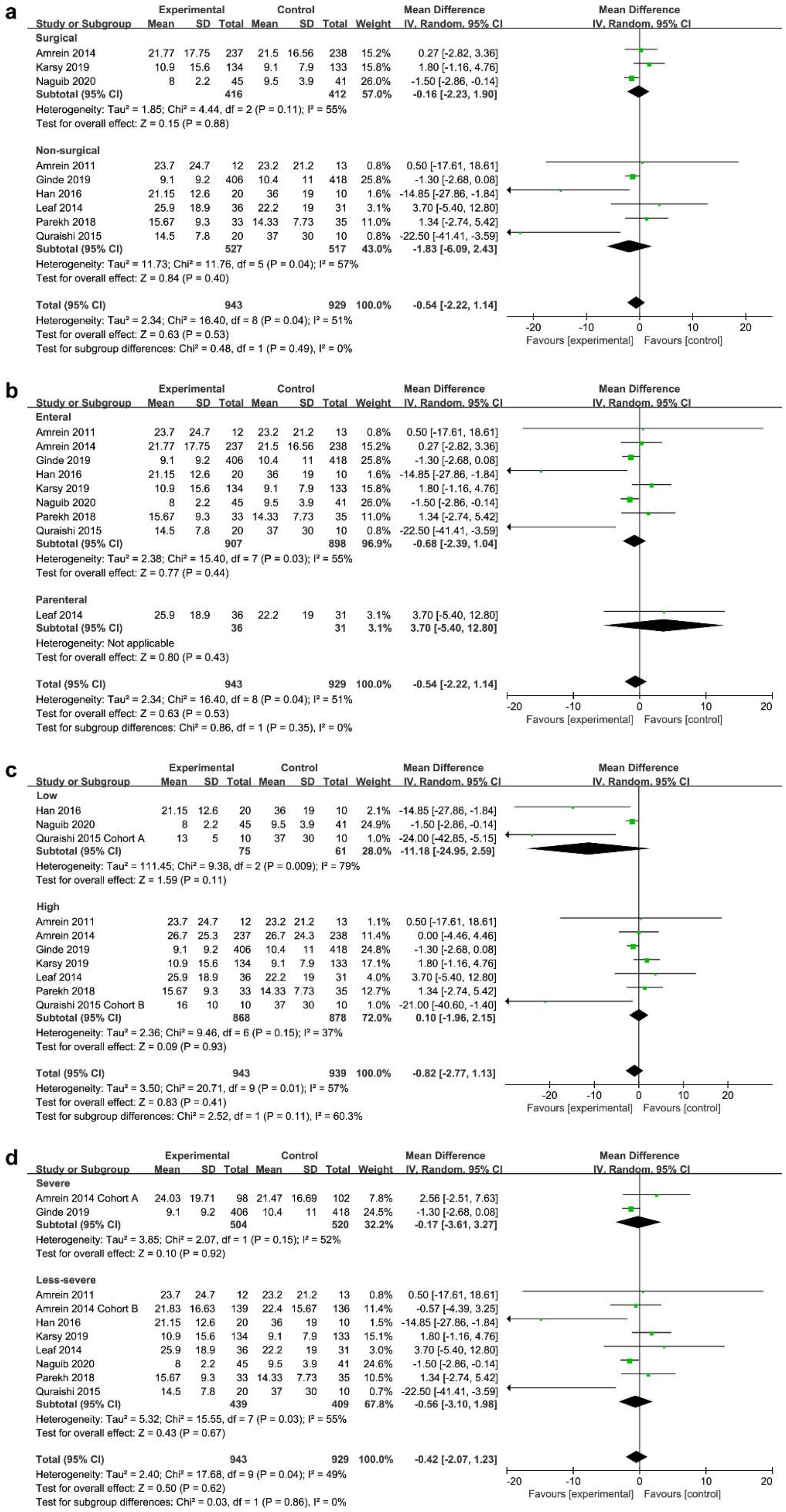

e-Figure 8: Subgroup analysis for length of hospital stay. (a) surgical patients versus non-surgical; (b) enteral route versus parenteral; (c) low dose of Vitamin D administration versus high; (d) severe Vitamin D deficiency versus less-severe

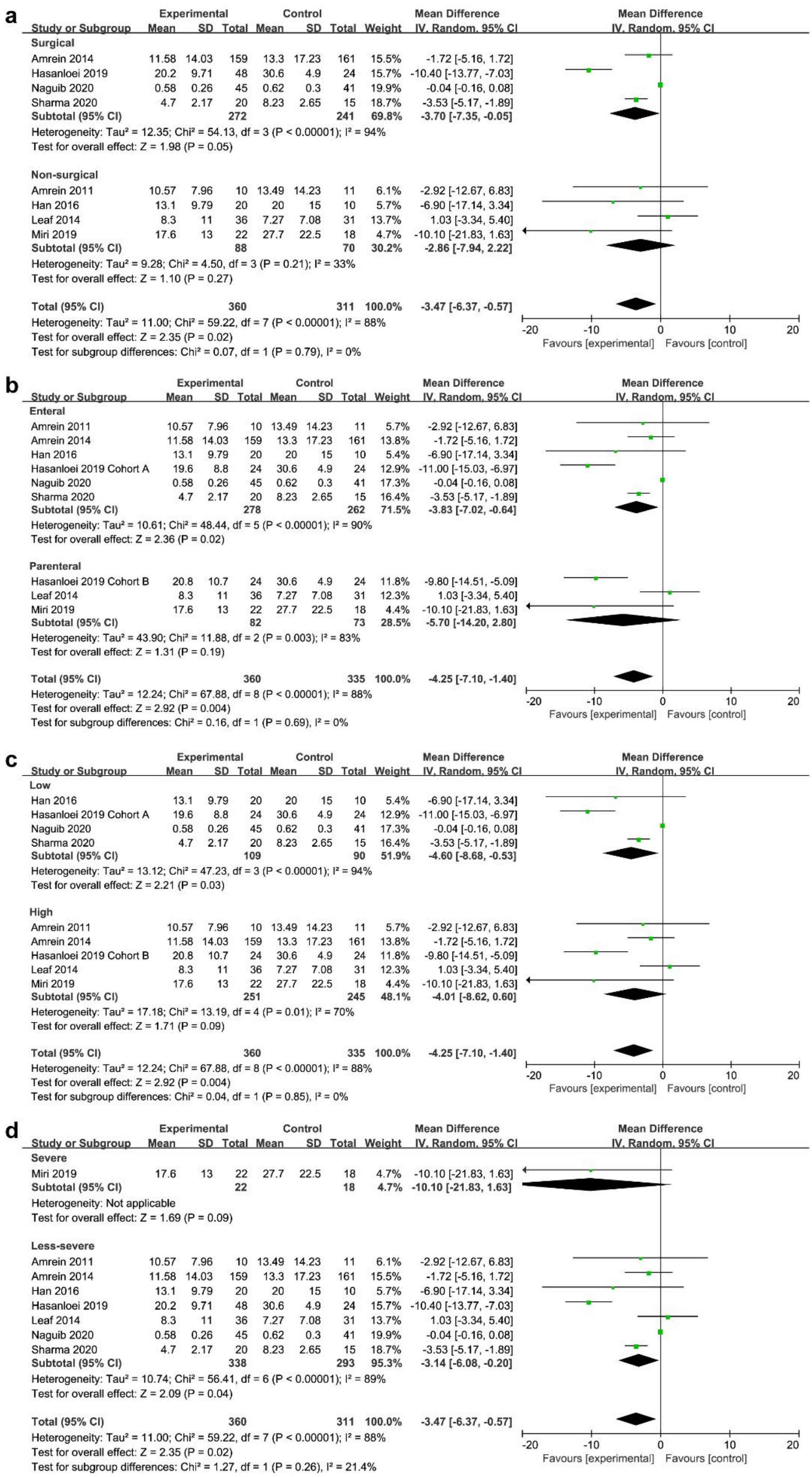

e-Figure 9: Subgroup analysis for duration of MV. (a) surgical patients versus non-surgical; (b) enteral route versus parenteral; (c) low dose of Vitamin D administration versus high; (d) severe Vitamin D deficiency versus less-severe

	Experimental		Control			Odds Ratio
Study or Subgroup	Events	Total	Events	Total	Weight	M-H, Random, 95% CI
Amrein 2014	159	237	177	238	68.4%	0.70 [0.47, 1.05]
Han 2016	5	20	3	10	3.8%	0.78 [0.14, 4.21]
Ingels 2020	9	11	11	13	2.3%	0.82 [0.10, 7.02]
Naguib 2020	16	45	23	41	14.4%	0.43 [0.18, 1.03]
Parekh 2018	13	33	12	35	11.1%	1.25 [0.46, 3.34]
**Total (95% CI)**		**346**		**337**	**100.0%**	**0.70 [0.51, 0.98]**
Total events	202		226			

Heterogeneity: Tau² = 0.00; Chi² = 2.54, df = 4 (P = 0.64); I² = 0%
Test for overall effect: Z = 2.10 (P = 0.04)

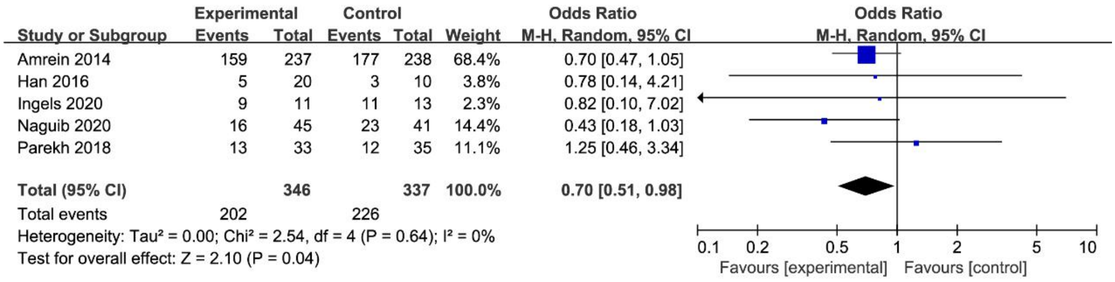

e-Figure 10: Effect of Vitamin D administration on infection in critically ill patients
